# Spillover benefit of pre-exposure prophylaxis for HIV prevention: evaluating the importance of effect modification using an agent-based model

**DOI:** 10.1017/S0950268822001650

**Published:** 2022-10-28

**Authors:** Ashley L. Buchanan, Carolyn J. Park, Sam Bessey, William C. Goedel, Eleanor J. Murray, Samuel R. Friedman, M. Elizabeth Halloran, Natallia V. Katenka, Brandon D. L. Marshall

**Affiliations:** 1 Department of Pharmacy Practice, College of Pharmacy, University of Rhode Island, Kingston, RI, USA; 2 Department of Epidemiology, Brown School of Public Health, Providence, RI, USA; 3 Department of Epidemiology, Boston University School of Public Health, Boston, MA, USA; 4 Department of Population Health, School of Medicine, New York University, New York, NY, USA; 5 Vaccine and Infectious Disease Division, Fred Hutchinson Cancer Center, Seattle, WA, USA; 6 Department of Biostatistics, University of Washington, Seattle, WA, USA; 7 Department of Computer Science and Statistics, College of Arts and Sciences, University of Rhode Island, Kingston, RI, USA

**Keywords:** Agent based models, causal inference, effect modification, HIV prevention, interference, network, pre-exposure prophylaxis, spillover

## Abstract

We developed an agent-based model using a trial emulation approach to quantify effect measure modification of spillover effects of pre-exposure prophylaxis (PrEP) for HIV among men who have sex with men (MSM) in the Atlanta-Sandy Springs-Roswell metropolitan area, Georgia. PrEP may impact not only the individual prescribed, but also their partners and beyond, known as spillover. We simulated a two-stage randomised trial with eligible components (≥3 agents with ≥1 HIV+ agent) first randomised to intervention or control (no PrEP). Within intervention components, agents were randomised to PrEP with coverage of 70%, providing insight into a high PrEP coverage strategy. We evaluated effect modification by component-level characteristics and estimated spillover effects on HIV incidence using an extension of randomisation-based estimators. We observed an attenuation of the spillover effect when agents were in components with a higher prevalence of either drug use or bridging potential (if an agent acts as a mediator between ≥2 connected groups of agents). The estimated spillover effects were larger in magnitude among components with either higher HIV prevalence or greater density (number of existing partnerships compared to all possible partnerships). Consideration of effect modification is important when evaluating the spillover of PrEP among MSM.

## Introduction

Men who have sex with men (MSM) remain a population at high risk for HIV infection in the United States (US) and face barriers to optimal use of HIV prevention modalities, such as pre-exposure prophylaxis (PrEP), particularly in the Southern US [1]. Many MSM are embedded in sexual risk networks such that the intervention may not only impact the individual prescribed PrEP, but also benefit their partners and beyond, which is known as *spillover* [2]. The magnitude and extent to which PrEP confers spillover benefits to persons not prescribed themselves remains poorly understood.

Current PrEP prescribing guidelines from the Centers for Disease Control and Prevention (CDC) focus primarily on individual HIV risk behaviours, missing features of the sexual networks and larger context [3, 4]. The package insert for brand-name PrEP medications does include assessment of HIV prevalence in individual's social networks and other factors that increase vulnerability, including incarceration, exchanging sex for commodities and drug use [5]. However, the impact of features of the sexual network on spillover effects of PrEP remains largely unknown.

Causal inference using a potential outcomes framework [6] was proposed to estimate spillover effects in agent-based models [7], which are a type of individual-based microsimulation. Due to the complexity of the exposures resulting in spillover and various assumptions about the spillover mechanism, identification and estimation of causal effects is critical to ensuring the validity of the estimated spillover effects [8]. Previous work employed an agent-based model to emulate a two-stage randomised trial to quantify spillover effects of PrEP use among MSM in Atlanta, Georgia (GA) [9]. That study demonstrated a spillover benefit of PrEP in the sexual networks of MSM among persons not assigned to PrEP themselves; however, this prior study did not consider effect modification. Effect modification of spillover effects is possible by component-level characteristics in a sexual network, such as HIV and drug use prevalence, which, if present, could be used to increase PrEP uptake and better allocate resources, such as increasing prescriber time with individuals whose treatment may benefit that individual and their partners [10]. Components are defined as subsets of agents (individuals) connected through sexual partnerships in a sexual network, but not sharing partnerships with agents in other components.

Nationally, African American (AA) MSM have an estimated HIV prevalence (25%) more than double that of White (W) MSM (8%) [11]. In Atlanta, GA, this difference was more pronounced with an estimated prevalence of 43% among AAMSM, compared to 13% among WMSM. Neither race-specific assortativity in sexual partnering nor sexual behaviours can fully explain these differences [12], highlighting that these disparities are driven by more distal, structural factors (like poverty and incarceration), rather than individual behaviours. Despite these disparities in HIV prevalence, PrEP uptake among AAMSM remains limited [13, 14]. Furthermore, several studies have demonstrated that the CDC criteria for prescribing PrEP poorly identified AAMSM who eventually seroconverted [4, 15]. Evaluation of effect modification offers information on what types of sexual networks could benefit the most from an intervention with spillover benefit.

In this methodologically-focused study that conducts causal inference with agent-based modelling, we evaluated effect measure modification of spillover effects by component-level characteristics, including HIV and drug use prevalence and network features, specifically density [16] and bridging potential [17], in the context of a simulated two-stage randomised trial. Bridging potential is a measure of centrality of an individual agent where they could act as a mediator between two or more closely connected groups of agents. We adapted a previously calibrated model of PrEP uptake and HIV transmission among MSM in the Atlanta metropolitan area to evaluate spillover effects of PrEP on the outcome HIV incidence [9, 18, 19]. We aimed to evaluate the magnitude and direction of possible spillover effects of PrEP use across predefined levels of the component-level effect modifiers to demonstrate the utility of causal inference methodology in agent-based modelling.

## Methods

### Model setting and simulated trial

We used the TITAN (Treatment of Infectious Transmissions through Agent-based Network) model [20] to simulate an agent-based model of PrEP uptake and HIV transmission among MSM in the Atlanta metropolitan area that was calibrated using available published data [12, 18, 19, 21]. We employed Latin hypercube sampling for model calibration [22]. Outputs from the initial model setting were obtained and compared to observed race-specific incidence rates from InvolveMENt [12, 23] (see Supplementary Appendix 7). This agent-based model included 17 440 agents to represent the estimated number of WMSM and AAMSM ages 18 to 39 years old living in the Atlanta-Sandy Springs-Roswell region [21, 24]. We then employed this calibrated model setting with demographics of this target population in our study; however, our aim was to simulate a randomised trial using this model, rather than recreate the HIV epidemic in this setting.

The agent-based model simulated a population of agents within a static sexual network; relationships and agents were initiated at population creation, and no agents entered or exited the model during the simulated trial for each run of the model with monthly time steps. Agents were assigned demographics, sexual behaviour characteristics, and HIV prevalence and treatment at model initiation, and components in the sexual network were identified based on sexual partnerships at model initialisation. Partner selection was a function of race and drug use class [25], which resulted in the generation of assortative sexual networks. This model used a ‘bottom-up’ approach for generating networks, where agents were assigned a number of partners, types of partners, then partnerships were selected through an iterative process [20]. At model initialisation for each model run, agents were assigned a target number of sexual partners. The total number of sexual partners per year was assumed to follow a negative binomial distribution with median = 5 for AAMSM and median = 7 for WMSM [12]. The number of sexual acts per month within a partnership was assumed to follow a Poisson distribution, and each agent was assigned a total number of sexual acts per monthly time step, based on a mean number of monthly number of sexual acts identified at model initialisation [26]. For each agent, a pool of potential partners was created from all other agents seeking partners, and subsequently narrowed by sexual position and the agent's assorting probabilities. An agent selected partners from this generated pool to achieve its target number of partners.

The per-act probability of HIV transmission in a serodiscordant sexual partnership was assumed to be constant for the duration for the two years and modified by the following factors: number of acts; condom use; type of anal intercourse; PrEP use and adherence (if HIV-negative and assigned); HIV infection status (acute *vs.* chronic), antiretroviral therapy (ART) use and adherence and viral suppression (if partner is HIV-positive). For serodiscordant partnerships with condomless sex, there were non-zero per-act probabilities of HIV transmission (per-act probability for condomless receptive anal intercourse was 1.38% and condomless insertive was 0.11%) [27]. At each monthly time interval, information on each agent and their component were recorded, including HIV status, ART use and viral load (if HIV-positive) and number of sexual acts.

We simulated a two-stage randomised design among MSM in Atlanta evaluating spillover effects of PrEP on the outcome cumulative HIV incidence by two years after randomisation [9, 18, 19]. Because we were interested in component-level effect measure modification, we required the components in the simulated trial to have at least three agents (at least one HIV+ agent) and individual agents had to be HIV-negative to be assigned to PrEP. These inclusion criteria ensured that the components included individuals at risk for HIV infection and the network structure of the component was complex enough to measure centrality.

In this simulated two-stage randomised design, enrolled components were first randomised 1:1 to either a PrEP allocation strategy (‘intervention’ components) or no PrEP allocation (‘control’ components). Then, eligible agents in each intervention component were randomised to PrEP according to a specific coverage level defined by the assigned allocation strategy. We considered the scenario of 70% PrEP coverage (on average) in intervention components to provide insight into strategies with high PrEP coverage.

We assumed the same set of parameters for this study as for the previously published work [21]. However, some key adaptations were made for this study [9]. We assumed static sexual networks defined at baseline and fixed for the study duration in each simulated trial. In this static sexual network, agents could have any number of partnerships with one or more sexual acts per monthly interval according to the initial model parameterisation (see Supplementary Table S1). The full sexual sociometric network was a set of smaller components, each agent belonged to only one component, and there were no partnerships between agents in different components. We also assumed no drop out (i.e. 100% retention in PrEP over the two years). These assumptions were necessary because the existing methods for evaluating spillover do not allow for time-varying components in the sexual network.

We added a ‘drug use’ agent class, which was defined at model initialisation and remained stable for the duration of the simulated trial. The prevalence of drug use was defined based on a review of relevant literature [28]. Drug use was defined as self-reported use of cannabis, cocaine, amphetamines, methamphetamines, inhalant nitrites, heroin/opioids or benzodiazepines in the past 12 months and influenced PrEP adherence, condomless sex and assortativity. Specifically, agents who were defined as using drugs had a 35% lower probability of adherence to PrEP [25] and 20% higher probability of condomless sex [29]. We assumed that 20% of drug-using agents mixed with other drug-using agents.

Python software, version 2.7.12, along with the NumPy and NetworkX packages, was used for coding, testing and performing sensitivity analyses of this model. The analysis of model output for this paper was generated using SAS software (version 9.4, Cary, NC, USA). R software, version 3.5.1, along with ggplot2, was used to produce figures. Additional information regarding parameter values, key model assumptions, data sources and additional references are included in the Supplementary Appendices 1–8 (Table S1).

### Causal inference methods for spillover

Let *M* be a baseline (pre-randomisation) binary component-level variable (1 = presence of a component-level factor, 0 = absence of that factor). Let *Y* denote the agent-level outcome ascertained two years after randomisation in the simulated trial. Given the two-stage randomised design, we expect exchangeability to hold at both the component and agent levels. We assume exchangeability within levels of *M*; that is, *Y*^*a*^⊥*A*|*M*. This means that the intervention components are comparable to control components, and within intervention components, agents randomised to the intervention are comparable to those randomised to the control, in expectation conditional on *M* at baseline [30].

Although static during follow-up, the component sizes at model initialisation varied in each simulated trial due to the partnering algorithm, so we extended estimators in [31] to evaluate effect modification by component characteristics. We assume *partial interference*; that is, an agent's outcome is influenced only by others in the same component, but no agents outside the component. We also assume *stratified interference*, in which an agent's potential outcome is dependent only on their own intervention assignment and the proportion of agents randomised to the intervention in their component [2]. We make the usual additional assumptions required for causal inference (i.e. exchangeability, consistency and positivity) [30]. We assumed a Bernoulli allocation strategy for intervention assignment within each component [2].

To evaluate effect modification by network features for the intention-to-treat effects, we first averaged agent-level characteristics for each component to determine the distribution across all components, then used this distribution to define a binary variable for each component. We are interested in quantifying a spillover effect, defined as the difference in the risk of HIV infection of an agent assigned to no PrEP under two different coverage levels of PrEP. This parameter can weight each agent or each component equally in the study population. We focused the presentation on the component-weighted estimators and compared to the individual-weighted estimators in the Supplementary Appendix 9 [30].

For the estimator, we computed the inverse probability weights conditional on *M*, then we estimated the causal effect within each level of the component-level variable. These weights are two-stage, inverse probability weights and correspond to the probability of intervention assignment at the component level and at the agent level conditional on the component intervention assignment. We employed both stabilised and unstabilised estimators in the analysis [31]. These estimators are unbiased in a two-stage randomised design with a single allocation strategy and a control group (no agents were assigned to PrEP) [31]. The estimators of the risk ratio of the spillover effect are defined analogously [31]. See Supplementary Appendix 12 for additional details on causal inference methods for effect modification of spillover.

### Network structure of components

The network features considered were the bridging potential and density. A component's *density* is defined as the proportion of observed connections in a component among the maximum number of possible connections in a component of the same size [16]. A sexual network component that is both large and dense is more likely to have agents who engage in sexual partnerships within the network component. This is particularly problematic when a pair of agents is HIV serodiscordant.

**Fig. 1.**
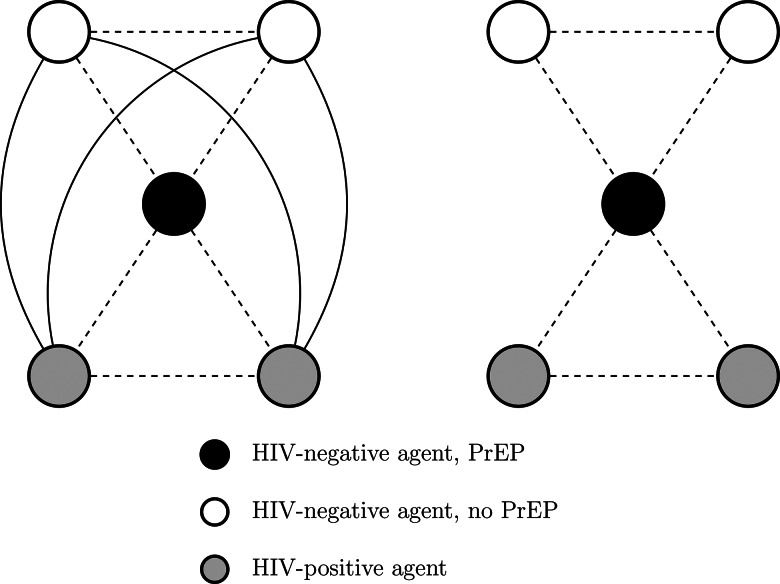
HIV-negative agent on PrEP who has no bridging potential (left) *vs.* high bridging potential (right). Dashed lines represent sexual partnerships present in both components, while solid lines represent sexual partnerships in the fully connected component only (left). Adapted from [32].

Bridging potential (also known as effective size) measures the redundancy in an agent's partnerships by examining the connections between their partners, providing a measure of centrality of an agent where they could act as a mediator between two or more closely connected groups of agents [17]. Bridging potential can be used to identify critical agents for interrupting HIV transmission chains in a network component. Agents with high bridging potential can act as ‘gatekeepers’ in the network [16] and, in the context of HIV, divide relatively isolated groups of other agents. If these agents remain uninfected, for example, by adhering to a PrEP regimen, they would limit or slow the spread of infection in the population (Fig. 1). Intervening to deliver PrEP to an HIV-negative agent with low bridging potential would only protect that agent against HIV acquisition; however, intervening to deliver PrEP to an HIV-negative agent with high bridging potential would protect that agent and the other HIV-negative agents in the component [32]. See Supplementary Appendix 13 for additional details on the network measures.

### Outcome measures

The primary outcome measure was cumulative HIV incidence over 24 months after randomisation, as measured by the number of incident HIV infections among those HIV-negative at the start of the trial (and reported as a proportion). We examined effect modification on both the ratio and difference scale using stabilised component-weighted estimators [31]. We estimated *spillover* effects within levels of the following component-level effect modifiers aggregated to the component level: HIV prevalence and drug use prevalence; and network characteristics: average density and average bridging potential. For each of the four effect modifiers, we defined binary variables based on the distribution of each variable (e.g. *M* = 0 if ≤median *vs. M* = 1 if >median). These parameters were computed using nonparametric estimators for this setting, averaging across 1000 simulations, along with 95% simulation intervals (SIs) (i.e. middle 95% of simulated output) to assess stochastic uncertainty [33].

### Sensitivity analyses

We conducted a sensitivity analysis in which PrEP coverage in the intervention components was set to 30% (on average), reflecting a lower coverage PrEP strategy. Furthermore, spillover is more meaningful if there are at least two HIV-negative agents in a component, so we conducted a sensitivity analysis excluding components with only one HIV-negative agent, which comprised about 13% of all components on average across the simulation runs. The model results may also depend on PrEP adherence and discontinuation among the agents randomised to PrEP. We performed one-way sensitivity analyses to assess the impact of our model parameterisation for PrEP adherence and PrEP discontinuation on model results for HIV cumulative incidence, focusing on two-stage randomised trials with 70% coverage allocation strategies in the intervention components. We quantified the effect measure modification, as specified above, across component-level factors. Specifically, we modified the proportion who were optimally adherent to PrEP in a monthly interval (i.e. 4 or more doses per week) to be 80% among WMSM and 50% among AAMSM [34]. We considered a scenario where 10% of agents discontinued PrEP in each monthly interval during the two-year follow-up [35].

## Results

In the two-stage simulated trial, there were an average of 3947 agents per simulation and about 800 components per trial with an average component size of 5 agents (standard deviation (s.d.) = 3). Characteristics of components were balanced between the intervention and control components (Table 1). On average, HIV prevalence was approximately 34% in each component at enrolment (95% simulation interval (SI) = 33%, 36%). About 45% of agents were AA (95% SI = 42%, 47%) and prevalence of drug use was 35% in the components (95% SI = 34%, 37%), on average. Average bridging potential was 1.51 (s.d. = 0.17); therefore, an agent in a network component typically has either a single connection or lies between two other agents. Average density was 0.49 (s.d. = 0.17), implying about half of the possible connections were made in the simulated network.

**Table 1.** Characteristics of components at the time of enrolment into the simulated two-stage randomised trial with 70% PrEP coverage in the intervention group in an agent-based model representing MSM in the Atlanta metropolitan area, Georgia, 2015–2017

		Component assignment	
Characteristics	Summary measure	Intervention (*n* = 1977.0)	Control (*n* = 1969.5)
Number of components		401.1	399.7
Average component size	Mean (s.d.)	4.9 (3.2)	4.9 (3.1)
	Median (IQR)	4 (2)	4 (2)
HIV prevalence		34.2%	34.3%
African American race		44.7%	44.8%
Any drug use		35.4%	35.4%
Bridging potential	Mean (s.d.)	1.5 (0.2)	1.5 (0.2)
	Median (IQR)	1.5 (0.3)	1.5 (0.3)
Density	Mean (s.d.)	0.5 (0.2)	0.5 (0.2)
	Median (IQR)	0.5 (0.3)	0.5 (0.3)

*Note*: Results above are from 1000 iterations of the agent-based model.

We evaluated the cumulative HIV incidence by 24 months of follow-up overall in the simulated trial and by four effect modifiers at the component level (Table 2). Overall, cumulative incidence among those in control components was 10% (95% SI = 8%, 12%), compared to 9% among agents randomised to no PrEP in the intervention components (95% SI = 6%, 12%) and 1% among agents randomised to PrEP in intervention components (95% SI = 0.5%, 2%). Among components with HIV prevalence above the median, the HIV cumulative incidence was about twice as high for all three groups compared to components with prevalence below the median. A similar but more modest effect modification was observed for density. For drug use and bridging potential, the effect modification was reversed only among agents not on PrEP in both intervention and control components and also attenuated compared to HIV prevalence.

**Table 2.** Cumulative incidence of HIV over two years of follow-up after two-stage randomisation stratified by four modifiers (≤median *M* = 0 *vs.* >median *M* = 1) among HIV-negative agents within PrEP intervention (70% coverage) and control components with 95% simulation intervals (SI) in an agent-based model representing MSM in the Atlanta metropolitan area, Georgia, 2015–2017 (*n* = 3947)

	Intervention components						Control components		
	Agents on PrEP			Agents not on PrEP			Agents not on PrEP		
Effect modifiers	Total agents	Incident HIV infections	Cumulative incidence^a^	Total agents	Incident HIV infections	Cumulative incidence^a^	Total agents	Incident HIV infections	Cumulative incidence^a^
Overall	909.8	10.4	0.01	389.7	35.4	0.09	1295.7	125.2	0.10
Drug use									
Among *M* = 0	490.6	6.4	0.01	210.2	21.1	0.10	698.9	74.3	0.11
Among *M* = 1	419.2	4.0	0.01	179.5	14.4	0.08	596.8	51.0	0.09
HIV Prevalence									
Among *M* = 0	700.1	6.0	0.01	300.1	21.6	0.07	998.0	77.5	0.08
Among *M* = 1	209.7	4.4	0.02	89.7	13.8	0.16	297.7	47.7	0.16
Bridging potential									
Among *M* = 0	352.4	4.9	0.01	150.3	16.4	0.11	501.0	57.3	0.11
Among *M* = 1	557.4	5.5	0.01	239.4	19.0	0.08	794.7	67.9	0.09
Density									
Among *M* = 0	719.3	7.5	0.01	308.1	25.8	0.08	1024.9	92.2	0.09
Among *M* = 1	190.5	2.9	0.02	81.5	9.6	0.12	270.9	33.0	0.12

a Cumulative incidence presented as a proportion (number of HIV+/total number).

Table 3 displays the estimated spillover effects of PrEP on cumulative incidence of HIV by 24 months on the ratio and difference scale stratified by the four binary modifiers. For the effect modifier drug use, the estimated spillover effects were slightly larger in magnitude among components with a lower prevalence of drug use compared to those with a higher prevalence. For bridging potential, the estimated spillover effects were larger among components with lower average bridging potential, compared to those with higher bridging potential.

**Table 3.** Estimated spillover effects of PrEP on cumulative incidence of HIV over two years of follow-up after two-stage randomisation stratified by four effect modifiers (≤median *M* = 0 *vs.* >median *M* = 1) among HIV-negative agents within PrEP intervention and control components with 95% simulation intervals (SI) in an agent-based model representing MSM in the Atlanta metropolitan area, Georgia, 2015–2017 (*n* = 3947)^a^

	RD (95% SI)	RR (95% SI)
*M* = 0		
Drug use	−0.08 (−0.14, −0.03)	0.47 (0.24, 0.78)
HIV prevalence	−0.05 (−0.10, −0.01)	0.56 (0.26, 0.91)
Bridging potential	−0.08 (−0.12, −0.03)	0.44 (0.23, 0.72)
Density	−0.06 (−0.10, −0.01)	0.58 (0.30, 0.94)
*M* = 1		
Drug use	−0.07 (−0.14, 0.001)	0.53 (0.20, 1.01)
HIV prevalence	−0.13 (−0.22, −0.04)	0.41 (0.18, 0.76)
Bridging potential	−0.04 (−0.03, 0.02)	0.68 (0.31, 1.21)
Density	−0.08 (−0.14, −0.02)	0.42 (0.17, 0.79)

RD, risk difference; RR, risk ratio.

a Estimates calculated using component-weighted stabilised estimators [31].

**Fig. 2.**
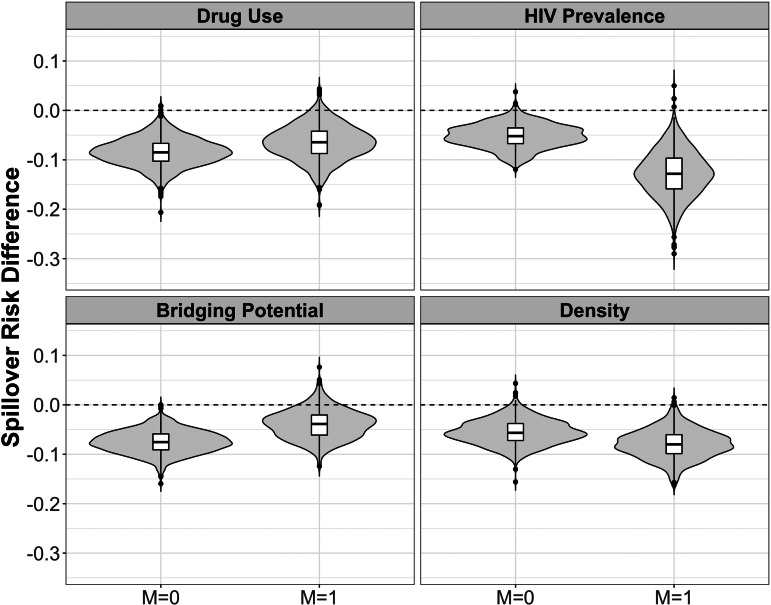
Estimated spillover risk difference of PrEP on cumulative incidence of HIV by effect modifiers. *M* = 1 if prevalence above median (*vs. M* = 0 at or below median) among HIV-negative agents within PrEP intervention (70% coverage) and control components in two-stage randomised designs of a PrEP intervention with 70% coverage in an agent-based model representing MSM in the Atlanta metropolitan area, Georgia, 2015–2017. Lines within boxes, median values; box borders, interquartile ranges (75th and 25th percentiles); bars, 90th and 10th percentiles; points, outliers. Shaded shape represented the distribution of estimates and dashed lines represent the null value (*n* = 3947).

**Fig. 3.**
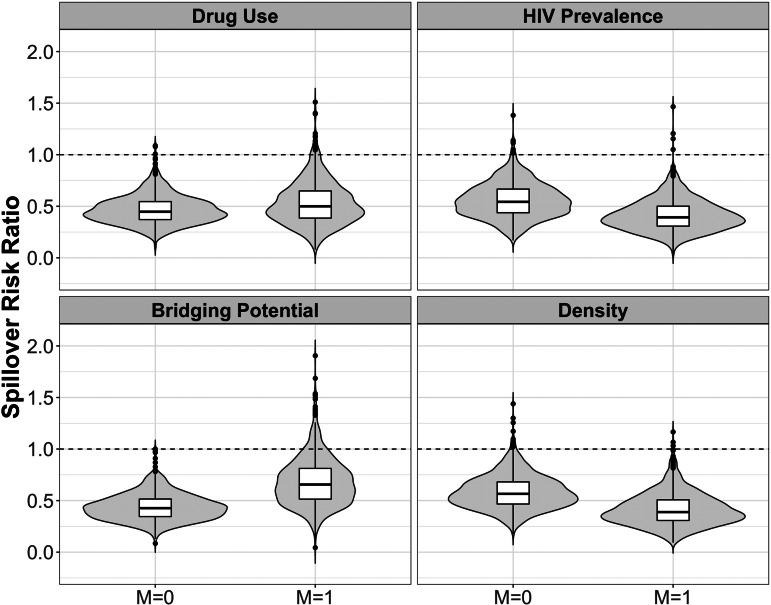
Estimated spillover risk ratio of PrEP on cumulative incidence of HIV by effect modifiers. *M* = 1 if prevalence above median *vs. M* = 0 at or below median among HIV-negative agents within PrEP intervention (70% coverage) and control components in two-stage randomised designs of a PrEP intervention with 70% coverage in an agent-based model representing MSM in the Atlanta metropolitan area, Georgia, 2015–2017. Lines within boxes, median values; box borders, interquartile ranges (75th and 25th percentiles); bars, 90th and 10th percentiles; points, outliers. Shaded shape represented the distribution of estimates and dashed lines represent the null value (*n* = 3947).

The estimated spillover effects were larger in magnitude among components with higher HIV prevalence compared to lower prevalence. Similar patterns were observed for network density although the effect modification was attenuated on the difference scale. Figures 2 and 3 display the estimated spillover effects across 1000 simulated trials on the difference and ratio scale. Consistent with the results in Table 3, the spillover effects among components with HIV prevalence above the median were larger in magnitude on both the difference and ratio scale, as compared to components with prevalence below the median.

For the sensitivity analysis of PrEP coverage, we simulated the trial with a 30% coverage of PrEP in the intervention components. The estimated spillover effects were typically larger in magnitude with 70% coverage compared to 30% coverage (Supplementary Appendix 9 and Tables S2–S6). For the sensitivity analysis that excluded components with only one HIV-negative agent, the estimated stabilised component-weighted effects on both the risk difference and ratio scales were somewhat attenuated towards the null, except for HIV prevalence among *M* = 0 and bridging potential among *M* = 1. However, the effect measure modification observed in the main analysis largely remained but was also attenuated (Supplementary Appendix 10, Tables S7–S9 and Figures S1–S2). For the one-way sensitivity analyses, the estimated spillover effects were fairly robust to changes in both PrEP adherence and PrEP discontinuation and the results from both of these sensitivity analyses were comparable to the main analysis (Supplementary Appendix 11, Tables S10–S21 and Figures S3–S6).

## Discussion

To leverage causal inference in agent-based models, we simulated a two-stage randomised trial to evaluate effect modification of PrEP spillover effects among MSM in the Atlanta metropolitan area, Georgia [18, 20]. Estimated spillover effects were slightly larger in magnitude among components with a lower prevalence of drug use and larger among lower average bridging potential. Interestingly, the spillover effects were larger in magnitude in components with *higher* HIV prevalence and also greater network density. In components with higher HIV prevalence, the estimated spillover effect was a 13 fewer HIV infections per 100 agents, as compared to only 5 fewer HIV infections per 100 agents among components with lower HIV prevalence.

When there are more HIV-infected agents in a component, HIV-uninfected agents have an increased risk of HIV acquisition, and thus benefit more by having partners who are on PrEP due to the reduction in HIV transmission risk from their partners' concurrent partners [36]. In fact, a component with no HIV-infected individuals at enrolment and no sexual risk connections outside the component has zero HIV risk, regardless of PrEP status, so these were excluded from our study. Furthermore, the estimated effects were stronger in denser components. When there are more partnerships in a component, there are more opportunities for PrEP to make a difference by preventing HIV seroconversion of agents [37]. In this model, drug use decreased PrEP adherence and condom use, as well as influenced the partnering algorithm. A lower prevalence of drug use likely resulted in less sexual risk behavior in a component possibly bolstering the spillover effect.

Particularly in the Southern US, uptake of PrEP services remains concerningly low among AAMSM [14]. Recent efforts in Atlanta, GA, to expand access to PrEP through the county health department are notable; however, initiating and adhering to PrEP remain a significant challenge for successful delivery among AAMSM [35]. Disparities in PrEP uptake may weaken the population-level impact on HIV incidence [19, 38]. Many evaluations of the efficacy and effectiveness of PrEP focus on an individual effect without consideration of the sexual risk network. With a better understanding of spillover effects and important effect modifiers, the delivery of PrEP interventions could be tailored to the most at-risk components, possibly mitigating disparities in PrEP uptake between WMSM and AAMSM. For example, network-based interventions could involve persons living with HIV referring their HIV-negative partners to PrEP. In fact, models such as the one employed here could be used to conduct preliminary evaluations of network-based PrEP interventions to inform subsequent delivery in the population [39].

This simulated trial approach has several limitations. To evaluate effect measure modification of spillover effects, we designed this study to include components both of meaningful size and at-risk for HIV. To employ existing causal inference methods, we assumed that the sexual network was static over time, which could lead to HIV prevalence saturation sooner (or later) then a setting with a dynamic sexual network. As such, this simulation does not reflect the true underlying sexual networks among MSM in the Atlanta metropolitan area and the simulated trial is not intended to recreate the HIV epidemic in this setting. This approach could also create less turnover in sexual partnerships for the duration of the study resulting in less overall HIV transmission compared to a dynamic sexual network with more frequent partnership changes. In future work, we plan to develop an approach that allows for assessment of spillover with networks updated over time and incorporate assortative mixing by age, which may be more important in a dynamic sexual network. We considered the entire sexual component to be an interference (i.e. spillover) set, which means that the intervention status of one agent in the component could possibly affect the outcomes of all other agents in the component. If a component is large or not well connected through sexual behaviors, this assumption may be dubious. A more realistic interference set might include an agent's sexual partners and their partner's partners and also allow for the use of the calibrated population in the simulated trial. Future work should include an evaluation of different interference sets, for example, by considering an agent's partners, known as nearest neighbours [40].

Careful consideration of effect modification of intervention effects with possible spillover is useful to inform the development of interventions that leverage network features. Although this study provides methodological insights into the design of two-stage randomised network trials with a sexual network ascertained only at baseline assuming the network is static for the duration of the study, allowing the network to be dynamic may result in a misspecified model in the analysis. Important extensions are required to understand real-world HIV transmission and to inform real-world PrEP allocation decisions, including simulation of dynamic sexual networks, along with more extensive calibration and validation studies. Persons not on PrEP may benefit from being in a network with higher PrEP coverage levels, and this benefit may be larger when component-level risk factors are more prevalent. Guidelines could encourage providers to ask about the PrEP status of sexual partners and encourage individuals on PrEP to recommend to others in their sexual network.

## Data Availability

Model code used to generate this data can be found at https://pph-collective.github.io/TITAN/. Complete reference for the TITAN model is S. Bessey, Mary McGrath, Maximilian King, & Carolyn Park. (2020, November 10). marshall-lab/TITAN: v1.2.4 (Version v1.2.4). Zenodo. http://doi.org/10.5281/zenodo.4266540. Data sets and post-modelling analysis code are available on Brown’s Digital Repository at https://doi.org/10.26300/thhj-6v09.

## Supplementary material

For supplementary material accompanying this paper visit http://doi.org/10.1017/S0950268822001650. click here to view supplementary material
