# Supplementary material for: Spillover benefit of pre-exposure prophylaxis for HIV prevention: evaluating the importance of effect modification using an agent-based model
Source: Epidemiol Infect. 2022 Oct 28;150:e192. doi: 10.1017/S0950268822001650 (PMC9723998; doi:10.1017/S0950268822001650)
Supplement: Supplementary file 1 [file S0950268822001650sup001.docx]

*Epidemiology and Infection*

**Spillover Benefit of Pre-Exposure Prophylaxis for HIV Prevention: Evaluating the Importance of Effect Modification using an Agent-Based Model**

Ashley L. Buchanan, Carolyn J. Park, Sam Bessey, William C. Goedel,

Eleanor J. Murray, Samuel R. Friedman, M. Elizabeth Halloran, Natallia V. Katenka, and Brandon D.L. Marshall

**SUPPLEMENTARY MATERIAL**

**Table of Contents**

[Supplementary Appendix 1 4](#_Toc109834290)

[Supplementary Appendix 2 4](#_Toc109834291)

[Supplementary Appendix 3 5](#_Toc109834292)

[Supplementary Appendix 4 6](#_Toc109834293)

[Supplementary Appendix 5 7](#_Toc109834294)

[Supplementary Appendix 6 8](#_Toc109834295)

[Supplementary Appendix 7 9](#_Toc109834296)

[Supplementary Appendix 8 9](#_Toc109834298)

[Supplementary Appendix 9 10](#_Toc109834299)

[Supplementary Appendix 10 11](#_Toc109834300)

[Supplementary Appendix 11 12](#_Toc109834301)

[Supplementary Appendix 12 13](#_Toc109834302)

[Supplementary Appendix 13 17](#_Toc109834303)

[Supplementary Table S1 19](#_Toc109834304)

[Supplementary Table S2 20](#_Toc109834305)

[Supplementary Table S3 21](#_Toc109834306)

[Supplementary Table S4 22](#_Toc109834307)

[Supplementary Table S5 23](#_Toc109834308)

[Supplementary Table S6 24](#_Toc109834309)

[Supplementary Table S7 25](#_Toc109834310)

[Supplementary Table S8 26](#_Toc109834311)

[Supplementary Table S9 27](#_Toc109834312)

[Supplementary Table S10. 29](#_Toc109834314)

[Supplementary Table S11 30](#_Toc109834315)

[Supplementary Table S12 31](#_Toc109834316)

[Supplementary Table S13 32](#_Toc109834317)

[Supplementary Table S14 33](#_Toc109834318)

[Supplementary Table S15 34](#_Toc109834319)

[Supplementary Table S16 35](#_Toc109834320)

[Supplementary Table S17 36](#_Toc109834321)

[Supplementary Table S18. 37](#_Toc109834322)

[Supplementary Table S19 38](#_Toc109834323)

[Supplementary Table S20 39](#_Toc109834324)

[Supplementary Table S21 40](#_Toc109834325)

[Supplementary Figure S1 41](#_Toc109834326)

[Supplementary Figure S2. 44](#_Toc109834327)

[Supplementary Figure S3.. 45](#_Toc109834328)

[Supplementary Figure S4 46](#_Toc109834329)

[Supplementary Figure S5 41](#_Toc109834330)

[Supplementary Figure S6 42](#_Toc109834331)

[References 47](#_Toc109834332)

**Supplementary Appendix 1: Model Specifications and Details**

Agent-based models (ABMs) are a class of computational models that simulate interactions of individual autonomous agents from which the system as a whole is evaluated. In most epidemiologic applications of ABMs, agents represent persons who are characterized by biological functions, positioning among other agents, exposures and disease states. Then, the model outputs are used to evaluate population-level outcomes. The Treatment of Infectious Transmission in Agent-Based Networks (TITAN) model (1) was adapted to simulate an idealized two-stage randomized trial evaluating effect measure modification by component-level factors of the spillover effects of randomized pre-exposure prophylaxis (PrEP) assignment among components of agents in a sexual network representing men who have sex with men (MSM) in the metropolitan area of Atlanta, Georgia (2, 3). The model described in this Supplement was adapted from an existing ABM evaluating equity-based PrEP on HIV incidence among MSM in the Atlanta area and complete details can be found in Goedel et al., 2020 (4-7).

**Supplementary Appendix 2: Parameter Data Sources and Agent Population**

The percentage of men who have sex with men in each of the thirty counties in the Atlanta-Sandy Springs-Roswell metropolitan statistical area (MSA) was estimated from the literature. Using weights for each county defined as the proportion of male-male households out of all households among counties with similar urbanicity in the American Community Survey (8), urbanicity-stratified estimates of the percentage of adult men who had sex with a man in the past year were derived from the National Health and Nutrition Examination Survey for each county (9). Using the Centers for Disease Control and Prevention’s Wide-Ranging Online Database for Epidemiologic Research (WONDER) (10), these percentages were then applied to population sizes estimates of the adult males in each county in the Atlanta-Sandy Springs-Roswell MSA to estimate the total number of MSM in each county (n=65,890). We assume that 26.5% of all MSM aged 18 years and older in the region are Black/African American and White MSM between 18 and 39 years old, corresponding to 17,440 MSM (38.9% Black/African American; 61.1% White). The model was initialized to generate a population of 17,440 agents representing all Black/African American (AA) and White (W) MSM between 18 and 39 years old residing in the Atlanta–Sandy Springs–Roswell MSA. For each model iteration, the base population was generated through a stochastic process based on the racial distributions and race-specific HIV prevalence in the population, in addition to behavioral and clinical state information for each agent. The model was updated in monthly time steps and the following characteristics were updated in each time step: target number of sex acts per month; HIV status; and, if agent is HIV positive, HIV infection status (acute versus chronic), utilization of antiretroviral treatment (ART), and viral suppression.

Given the two years of follow-up in the simulated trial and the age distribution of the population, we assumed that there was no mortality and complete retention during follow-up. These assumptions were necessary because the existing methods for evaluating spillover do not allow for time-varying components (or spillover sets) in the sexual network. If the sexual network was allowed to be dynamic, the models used in the analysis may be misspecified. Several key parameters were stratified by race, including HIV prevalence, and engagement with the HIV treatment cascade. Prevalence of any drug use was also stratified by race (30% among African Americans, 49% among whites) (11-13). In addition, pre-enrollment HIV prevalence was also stratified by race (43% among African Americans, 13% among white) (14). Proportion with an HIV diagnosis, proportion on ART, and proportion with viral suppression (among those on ART) were also stratified by race (Supplementary Table S1). At model initialization, agents were assigned an HIV status. HIV-infected agents were then assigned the following: HIV infection status (acute versus chronic), utilization of ART, adherence to ART at model initialization, and achievement of viral suppression. Only HIV-negative agents were eligible to be randomized to PrEP in the simulated trial.

**Supplementary Appendix 3: Network Structure and Sexual Partnership Formation**

An agent’s race and drug use status affected their sexual partnering preferences. Any drug use was defined as self-report of use of cannabis, cocaine, amphetamines, methamphetamines, inhalant nitrites, heroin/opioids, or benzodiazepines in the past 12 months (14). For drug use, we assumed that 20% of drug using agents partnered only with other drug using agents. We assumed that the probability of same-race partnerships was 76.5% for AAMSM and 72.2% for WMSM (15).

All agents were assigned a sexual position preference (exclusively insertive, exclusively receptive, and versatile) at model initialization. The model included an absolute prohibition, such that two exclusively insertive agents could not pair with one another nor could two exclusively receptive agents. These parameters are derived from reports of role in last sex for up to 5 recent sexual partners as ascertained from the InvolveMENt cohort (14).

A sexual network was generated between agents using a “bottom-up” approach informed by the parameterization of sexual partnerships. At model initialization for each model run, agents were assigned a target number of sexual partners and sex acts per month (16-18). The total number of sexual partners per year was assumed to follow a negative binomial distribution with median = 5 for AAMSM (interquartile range (IQR): 3-10) and median = 7 for WMSM (IQR = 4-15) (11). A target partner number was determined based on the total number of target partners. The negative binomial is an appropriate distribution due overdispersion of the distribution of number of sexual partnerships in this population. The number of sexual acts per month within a partnership was assumed to follow a Poisson distribution, and each agent was assigned a total number of sexual acts per monthly time step (one or more acts) based on a mean number of monthly number of sexual acts identified at model initialization (14, 19).

Partnering occurred at model initiation, prior to trial enrollment, and the sexual network remained static at all subsequent timepoints in the simulated trial. These partnerships at enrollment were used to determine network components with at least three agents and at least one HIV-positive agent. For each agent, a pool of potential partners was created from all other agents seeking partners, and subsequently narrowed by sexual position and the agent’s assorting probabilities. The agent then selects partners from this generated pool to achieve its target number of partners. A network component is a subset of the agents of a network that are all connected through at least one partnership and not connected to any other agents in the network. Once formed, relationships were *not* dissolved and new relationships were *not* formed, but rather sexual networks were to be static, as initiated at population creation and ascertained prior to enrollment in the simulated trial (20).

**Supplementary Appendix 4: HIV Transmission**

Any two agents sharing a partnership (or edge) in the network could engage in anal intercourse. At each monthly interval, agents had a mean number of sex acts with their partners identified at model initiation, forming the mean of a Poisson distribution from which monthly sex acts were drawn. Sex acts using condoms were considered non-transmitting acts and therefore had no probability of HIV transmission. The base probabilities of condom use were informed by a cross-sectional assessment of condom use experiences as ascertained in the InvolveMENt cohort (21). Agents who used drugs had a 20% higher probability of condomless sex (22). In the absence of any HIV intervention (PrEP or ART for treatment as prevention), any condomless sex act in a serodiscordant partnership had a non-zero probability of HIV transmission (per-act probability for condomless receptive anal intercourse was 1.38% and condomless insertive was 0.11%) (23). For serodiscordant partnerships with condomless sex, there was a non-zero per-act probabilities of HIV transmission, which depended on number of acts, condom use, type of anal intercourse, PrEP use (for HIV-negative agents assigned to PrEP) (23). For example, there was a 96% reduction in the risk of HIV acquisition for agents fully adherent to PrEP and a 76% reduction for those with less than full adherence (24). In addition, HIV infection status (acute versus chronic), ART utilization, adherence to ART at model initiation, and viral suppression also modified the per-act probability of HIV transmission (25).

Once the per-act probability of transmission in a relationship was determined, the overall risk for the timestep was calculated and used to determine whether HIV transmission occurs in the serodiscordant partnerships. Agents using antiretroviral therapy but not in viral suppression had 19% reduction in the likelihood of HIV transmission to serodiscordant partners (26). Agents that were virally suppressed had no ability to transmit; however, this model did not have risk reduction for diagnosed agents. Given the low prevalence of injection risk behavior in this population, needle-sharing acts of injection drug use were not modeled.

**Supplementary Appendix 5: HIV Testing, Treatment and Disease Progression**

Based on the InvolveMENt cohort, HIV prevalence was 43.4% for AAMSM and 13.2% for WMSM (11). Based also on the InvolveMENt cohort, we assumed that 89.2% of AAMSM and 94.2% of all WMSM were ever tested for HIV infection. In subsequent time steps, 66.3% of AAMSM and 73.6% of WMSM were tested each calendar year. AAMSM obtained testing randomly with a monthly probability of 0.055 and WMSM obtained testing randomly with a monthly probability of 0.061. HIV-infected agents unaware of their infection tested at the same probability at HIV-uninfected agents. No restriction on re-testing within a year was imposed (i.e., an agent may test multiple times per year).

Based on (25), we assumed that 65.5% of African American MSM and 81.8% of White MSM with HIV were diagnosed at model initialization. Once diagnosed, HIV-infected agents may initiate ART and become virally suppressed. Based on (25, 27), we assumed that 65.8% of AAMSM and 59.7% of WMSM who were diagnosed then initiated ART (representing 43.1% of all African American agents with HIV infection and 48.8% of all White agents with HIV infection). At model initialization, the proportion of diagnosed HIV-infected agents achieving optimal adherence (i.e., taking 90% or more of all doses) to ART were informed by HIV care continuum surveillance activities. We assumed that 81.7% of white agents on ART were fully adherent to ART (43.2% of all white agents with HIV infection), and 88.5% of African American agents on ART were fully adherent to ART (35.2% of all African American agents with HIV infection) (25). We assumed adherence to ART was constant once agents initiate therapy and ART monthly discontinuation probability was 2.0% for AAMSM and 1.9% for WMSM (27). For viral suppression, we assumed that agents who have optimal adherence to ART will have viral load < 200 copies/mL.

After seroconversion, agents have an acute stage of infection lasting two months with an increased base per-act probabilities of HIV infection increased by a factor of 7.25 (11). After this period, agents are in a chronic state of HIV infection. Based on surveillance estimates for the state of Georgia reported by the National Center for HIV/AIDS, Viral Hepatitis, STD, and TB Prevention (NCHHSTP) of the Centers for Disease Control and Prevention, 51.7% of AAMSM and 53.1% of WMSM agents living with HIV were assumed to have AIDS. For progression to AIDS, a monthly probability was assigned to all HIV-infected agents based on their antiretroviral therapy and viral suppression. For HIV-infected agents not using antiretrovirals, the probability was 0.0029. Among agents using antiretrovirals, the probability was 0.0021 among agents who did not achieve viral suppression and 0.0009 among agents who achieved viral suppression (28). HIV-infected agents with suboptimal adherence to ART had identical probabilities of progression to AIDS as all other HIV-infected agents (regardless of their diagnosis status).

**Supplementary Appendix 6: Pre-Exposure Prophylaxis Use and Clinical Care**

In our simulated two-stage randomized trial, components within the sexual network were defined prior to enrollment in the trial among all MSM (ages 18 to 39 years); however, only components with at least one HIV-positive agent and three or more agents were eligible to become “intervention components,” and at the individual level, only HIV-negative agents with one or more partners were eligible to be enrolled in the trial and possibly randomized to PrEP. Depending on the allocation strategy assigned to the component, eligible agents were randomized to PrEP (or no PrEP). After enrollment in the trial, eligible agents who were randomized to the PrEP intervention received a 90-day supply, and then received a subsequent 90-day supply at each scheduled follow-up visit every 3 months. For the two-year duration of this simulated trial, all agents were retained in the study and partnerships remained constant. After enrollment in the trial, agents were classified as adherent (defined as 4 or more doses per week) or suboptimal (defined as 2 to 4 doses per week). In the main model, 91.1% of WMSM and 56.8% of AAMSM were considered to be optimally adherent to PrEP at baseline (i.e., 4 or more doses per week) (29-31). Those with optimal adherence had a 96% reduction in the per-act probability of HIV acquisition, while those with partial adherence had a 76% reduction (24). This remained stable for an individual agent across all model runs. Drug use also influenced PrEP adherence, specifically agents who used drugs had a 35% lower probability of adherence to PrEP (32). Agents discontinued PrEP only if they became infected with HIV.

**Supplementary Appendix 7: Model Calibration**

Model calibration was conducted using data from the InvolveMENt study. The primary calibration targets were reproduced using this model, specifically the race-specific incidence rates from InvolveMENt (6.5 per 100 person-years for Black/African American MSM and 1.7 per 100-person-years for White MSM) (11). Calibration was performed using Latin hypercube sampling, which samples from and searches a multidimensional parameter space (33). Complete details and results of the calibration procedure for this model have been previously published (7). For each set of parameters, the model was run to estimate a statistic reflecting how closely the set of parameters aligned with the empirical calibration targets. The calibration process focused on the parameters with the most uncertainty or those drawn from studies outside the target population. A set of scaling factors were used for the number of sex acts per month, per-month probability of being diagnosed following infection, and the per-month probability of initiating antiretroviral treatment after diagnosis, and the latter two were stratified by agent race. These scaling factors were used to first simulate stable proportions of agents in each stage of the HIV care cascade for the model duration and then to calibrate HIV incidence to align with the race-specific calibration targets.

**Supplementary Appendix 8: Technical Details**

Python software, version 2.7.12 (34), along with the NumPy (35) and NetworkX (36) packages, was used for coding, testing, and performing sensitivity analyses of this model. The analysis of model output for this paper was generated using SAS software (version 9.4). Copyright © 2021 SAS Institute Inc. SAS and all other SAS Institute Inc. product or service names are registered trademarks or trademarks of SAS Institute Inc., Cary, NC, USA. R software, version 3.5.1 (R Foundation for Statistical Computing, Vienna, Austria) (37), along with ggplot2 (38), was used to produce figures. At each monthly time interval, variable information on each agent and their component members were recorded, including HIV status, awareness of status, utilization of ART and viral suppression (if HIV positive), and number of sexual acts. Agents were assigned characteristics at model initiation and components were determined prior to enrollment in the simulated trial based on sexual partnerships formed at model initialization.

The simulations were run on Oscar, Brown University’s supercomputing cluster. Oscar operates on the CentOS 6.7 Linux operating system and utilizes the SLURM workload manager. The simulations were processed using 2.53 GHz Intel Xeon E5540 processors operating with 8 cores at 14.84 teraflops and 12GB of DDR3 memory. The model was run for 24 monthly time steps (2 years), included 17,440 agents total per model run, and averages over a total of 1,000 Monte Carlo runs per scenario, each with a simulated population based on the model parameters described above.

**Supplementary Appendix 9: Sensitivity Analyses for Intervention Coverage and Estimators**

For the sensitivity analysis, we simulated a trial with 30% coverage of PrEP in the intervention components, as well as the 70% coverage scenario. For each of the four effect modifiers,

we defined binary variables based on the distribution of each variable

(e.g., $M=0$ if $\leq$ median versus $M=1$ if $>$ median). There was an average of 3,911 agents per simulation in the simulated trial with the 30% coverage scenario and an average of 3,947 agents per simulation in the simulated trial with the 70% coverage. For the 30% coverage scenario, the cumulative incidence of HIV over 24 months in the simulated trial and stratified by each effect modifier are displayed by intervention and control components in Supplementary Table S2. We estimated the spillover effects of PrEP on HIV incidence by each effect modifier using individual- and component-weighted estimators (both stabilized and unstabilized) for the risk difference and risk ratio (Supplementary Tables S3 to S6). In general, the estimated spillover effects were larger in magnitude with 70% intervention coverage, as compared to 30% coverage. The results for the stabilized estimators were comparable to the unstabilized; however, there were some slight differences between the individual-weighted and component-weighted estimators. Interestingly, the trends observed in the main analysis with 70% coverage (e.g., larger in magnitude estimated spillover effects among components with a higher prevalence of HIV compared to components with a lower HIV prevalence) remained in this sensitivity analysis.

**Supplementary Appendix 10: Sensitivity Analyses among Components with Two or more HIV-negative Agents**

Spillover is more meaningful if at least two HIV-negative agents in a component, so we conducted a sensitivity analysis that excluded components with only one HIV-negative agent, which comprised about 13% of all components across the simulation runs. After excluding components with only one HIV-negative agent, PrEP coverage remained at 70% on average among intervention components and an average of 3,596 agents per simulation. Supplementary Table S7 displays the characteristics of the components in the sensitivity analysis. Compared to the main analysis, there were fewer components with a slightly larger average component size, with a slightly lower prevalence of HIV. The cumulative incidence of HIV over 24 months in the simulated trial and stratified by each effect modifier are displayed by intervention and control components in Supplementary Table S8. The cumulative incidence both overall and across levels of the effect modifiers was comparable between the main analysis and this sensitivity analysis. We estimated the spillover effects of PrEP on HIV incidence by each effect modifier using individual- and component-weighted estimators (both stabilized and unstabilized) for the risk difference and risk ratio (Supplementary Table S9 and Supplementary Figures S1 and S2). Comparing the stabilized component weighted estimators to the main analysis, the estimated effects on both the risk difference and ratio scales were somewhat attenuated towards the null, except for HIV prevalence among $M=0$ and bridging potential among $M=1$. However, the effect measure modification observed in the main analysis largely remained but was attenuated in the sensitivity analysis for the component-weighted stabilized estimators. The estimated spillover effects were slightly larger in magnitude among those components with a lower prevalence of drug use compared to higher drug use. For bridging potential, the estimated spillover effects were larger in magnitude only on the ratio scale among components with lower average bridging potential, compared to those with higher bridging potential. The estimated spillover effects were larger in magnitude only on the difference scale among components with higher HIV prevalence compared to lower prevalence, although this difference was somewhat attenuated compared to the main analysis. For density, the estimated spillover effects were larger in magnitude on the ratio scale only for components that had higher density compared to components with lower density. In the sensitivity analysis, the results for the stabilized estimators were comparable to the unstabilized; however, there were some slight differences between the individual-weighted and component-weighted estimators.

**Supplementary Appendix 11: One-Way Sensitivity Analyses**

We performed one-way sensitivity analyses to assess the impact of our model parameterization on model results for HIV cumulative incidence. We focused our reporting on two-stage randomized trials with 70% coverage allocation strategies in the intervention components because this was our primary model scenario. For the sensitivity analysis of PrEP adherence, there was an average of 3,967 agents per simulation in the simulated trial with the 70% coverage. For the sensitivity analysis of PrEP discontinuation, there was an average of 3,896 agents per simulation in the simulated trial with the 70% coverage. We used the component-weighted stabilized estimators in this sensitivity analysis. This sensitivity analysis evaluated the model results while varying assumptions about adherence to PrEP and discontinuation of PrEP. In the main model, 91.1% adherence for WMSM and 56.8% for AAMSM were considered to be optimally adherent to PrEP at baseline (i.e., 4 or more doses per week). In the sensitivity analysis, we considered 80% PrEP adherence for WMSM and 50% PrEP adherence for AAMSM. In the main model, agents only discontinued PrEP during follow-up if they HIV seroconverted, and in the sensitivity analysis, 10% of agents discontinued PrEP in each monthly interval (8).

In Supplementary Tables S10 to S13, we display the HIV prevalence and HIV cumulative incidence at the end of two years of follow-up after randomization based on a simulated trial with 70% coverage with 80% PrEP adherence for WMSM and 50% PrEP adherence for AAMSM. In most cases, the sensitivity analysis resulted in slightly more agents on PrEP with incident HIV, while also slight increases in HIV incidence among agents in intervention components but not on PrEP. In Supplementary Tables S14 to S17, we display the HIV prevalence and HIV cumulative incidence at the end of two years of follow-up after randomization based on a simulated trial with 70% coverage with 10% discontinuation of PrEP. In most cases, the sensitivity analysis resulted in substantially more agents on PrEP with incident HIV, while also small or no changes HIV incidence among agents in intervention components but not on PrEP.

Supplementary Table S18 to S21 display the estimated spillover risk differences (RD) and risk ratios (RR) for HIV cumulative incidence in a two-stage randomized trial with 70% coverage. In the main analysis, 91.1% adherence for WMSM and 56.8% for AAMSM were considered to be optimally adherent to PrEP at baseline (i.e., 4 or more doses per week). In the sensitivity analysis, we considered 80% PrEP adherence for WMSM and 50% PrEP adherence for AAMSM. Interestingly, the estimated spillover effects were fairly robust to this one-way sensitivity analysis for PrEP adherence and the results were comparable to the main analysis. Supplementary Figure S3 and Supplementary Figure S4 display the estimated spillover effects when PrEP adherence was lower in a simulated trial with 70% PrEP coverage on the difference and ratio, respectively.

Supplementary Table S18 to S21 display the estimated spillover risk differences (RD) and risk ratios (RR) for HIV cumulative incidence in a two-stage randomized trial with 70% coverage. In the main analysis, we assumed that no agents discontinued PrEP. In the sensitivity analysis, we considered a scenario where 10% of agents discontinued PrEP. Interestingly and despite increases in HIV incidence among those on PrEP, the estimated spillover effects were fairly robust to this one-way sensitivity analysis for PrEP discontinuation and the results were comparable to the main analysis. Supplementary Figure S5 and Supplementary Figure S6 display the estimated spillover effects when PrEP discontinuation was higher (10%) in a simulated trial with 70% PrEP coverage on the difference and ratio, respectively.

**Supplementary Appendix 12: Causal Inference Methods for Evaluation of Effect Modification in the Presence of Spillover in an ABM**

In each simulation, we employed two-stage randomization, which is a randomized trial design that allows for quantifying intervention effects in the presence of spillover (i.e., interference or dissemination). Spillover is when one agent’s intervention assignment affects another agent’s outcome. In a two-stage randomized design, components are first randomized to the intervention (e.g., 70% coverage of PrEP) or control (e.g., 0% coverage of PrEP), then according to the component-level allocation strategy, agents are randomly assigned to the intervention. In each run of the model, the component sizes vary, so we extended estimators from Basse and Feller (2018) to consider effect modification by component characteristics (39, 40). We assumed partial interference. That is, we assume that the intervention assignment influences others in the same component only; however, this influence does not extend beyond the component (41). We also assume *stratified interference* where an individual’s potential outcome is dependent only on their own intervention assignment and the proportion of agents assigned to PrEP in the component (42). We also make the usual assumptions required for causal inference (exchangeability, consistency, and positivity) (43-46). We assume a Bernoulli allocation strategy for intervention assignment within each intervention component (42).

There are $I$ components total and each of the component has $n_{i}$ individuals with $j=1,2,...,n_{i}$ and $\sum_{i=1}^{I} n_{i}=N$. Let $Y_{ij}, A_{ij}$ represent an observed outcome and treatment assignment status of $j^{th}$ agent in component $i$, respectively. Let $C_{i}$be an indicator for the treatment assignment at the component level. Let $M_{i}$ be a component-level variable measured at baseline (e.g., covariates aggregated to the component level or network characteristic of the component). We estimated the spillover effect within levels of the following component-level effect modifiers aggregated to the component level: HIV prevalence and drug use prevalence; and network characteristics: average density and average bridging potential (47-49). For now, we consider only categorical versions of the effect modifier $M_{i}$. We consider the potential outcome for agent $j$ in component $i$ as $Y_{ij}(\boldsymbol{A}_{i})=Y_{ij}(C_{i}=c, A_{ij}=a)$. Because we have a control group with no agents assigned to PrEP, there are three possible combinations of the intervention resulting in three potential outcomes: $Y_{ij}(1,1), Y_{ij}(1,0), Y_{ij}(0,0)$. The observed outcome is a function of the intervention assignment and potential outcomes; that is, $Y_{ij}^{obs}=C_{i}A_{ij}Y_{ij}\left( 1,1 \right)+C_{i}\left( 1-A_{ij} \right)Y_{ij}\left( 1,0 \right)+(1-C_{i})Y_{ij}(0,0).$Let $T_{ca}=\{(i,j): C_{i}=c and A_{ij}=a\}$to denote the set of components and agents who are assigned to $C_{i}=c$ and $A_{ij}=a$.

In the setting with varying component sizes, there are two types of estimands: component-weighted estimands that assign equal weight to components, regardless of the number of individuals in each component; and individual-weighted estimands that assign equal weight to individuals, regardless of the distribution across components (39). The spillover (i.e., disseminated or indirect) effect is $IE=\sum_{i=1}^{I} w_{i}^{*}\sum_{j=1}^{n_{i}} [Y_{ij}\left( 1,0 \right)-Y_{ij}\left( 0,0 \right)]$, where $w_{i}^{*}=\frac{1}{In_{i}}$ corresponds to component-weighted estimands and $w_{i}^{*}=\frac{1}{N}$corresponds to individual-weighted estimands with $N=\sum_{i} n_{i}$.

To estimate the spillover effect, we employ the two-stage inverse probability weights ${w_{i}}^{(00)}$, ${w_{i}}^{(10)}$, ${w_{i}}^{(11)}$ as ${w_{i}}^{(11)}=\frac{1}{Pr(C_{i}=1)}\frac{1}{Pr({A_{ij}=1|C}_{i}=1)}$, ${w_{i}}^{(10)}=\frac{1}{Pr(C_{i}=1)}\frac{1}{Pr({A_{ij}=0|C}_{i}=1)}$, and ${w_{i}}^{(0)}=\frac{1}{Pr(C_{i}=0)}$. Also define ${w_{i}}^{(c)}=\frac{1}{Pr(C_{i}=c )}$. The weighted spillover effect estimator is:

$\hat{IE}=\sum_{(i,j)\in T_{10}} {{w_{i}^{*}w}_{i}}^{(10)}Y_{\mathrm{ij}}^{obs}(1,0)-\sum_{(i,j)\in T_{00}} {{w_{i}^{*}w}_{i}}^{\left( 00 \right)}Y_{\mathrm{ij}}^{obs}(0,0)$.

These are unbiased estimators in a two-stage randomized design with one intervention allocation strategy and one control condition (39).

We define the effect measure modification parameters as follows, modestly extending results in (32). We define these on the difference scale below, but will also consider both the relative and absolute scales when analyzing the agent-based model results. Let the indicator function $I\left( M_{i}=m \right)=1$ if $M_{i}=1$ and $I\left( M_{i}=m \right)=0$, otherwise. Let $I_{m}$denote the number of components with $M_{i}=m$ and $n_{m}^{+}=\sum_{i} n_{i}I(M_{i}=m)$ denote the number of individuals in stratum $M_{i}=m$. Among components with $M_{i}=m$, the spillover (i.e., indirect) effect is

$IE_{m}=\sum_{i=1}^{I} w_{mi}^{*}\sum_{j=1}^{n_{i}} [Y_{ij}\left( 1,0 \right)-Y_{ij}\left( 0,0 \right)]$,

where $w_{mi}^{*}=\frac{1}{I_{m}n_{i}}$ corresponds to component-weighted estimands and $w_{mi}^{*}=\frac{1}{n_{m}^{+}}$corresponds to individual-weighted estimands.

To quantify the spillover effect modified by $M$, we proposed the following modifications to the two-stage inverse probability weights to allow for estimation of effect measure modification: ${w_{i}}^{(m11)}=\frac{1}{Pr(C_{i}=1|M_{i}=m)}\frac{1}{Pr({A_{ij}=1|C}_{i}=1,M_{i}=m)}$, ${w_{i}}^{(m10)}=\frac{1}{Pr(C_{i}=1{|M}_{i}=m)}\frac{1}{Pr({A_{ij}=0|C}_{i}=1,M_{i}=m)}$, and ${w_{i}}^{(m00)}=\frac{1}{Pr(C_{i}=0|M_{i}=m)}$. Also define ${w_{i}}^{(mc)}=\frac{1}{Pr(C_{i}=c |M_{i}=m)}$. We also revised the individual- and component-level weights: $w_{mi}^{*}=\frac{1}{I_{m}n_{i}}$ corresponds to component-weighted estimands and $w_{mi}^{*}=\frac{1}{n_{m}^{+}}$corresponds to individual-weighted estimands. Within each level of effect modifier $M,$ the weighted spillover effect estimator is

$$\hat{IE}_{m}=\sum_{(i,j)\in T_{10}} {{{{I(M}_{i}=m)w}_{mi}^{*}w}_{i}}^{(m10)}Y_{\mathrm{ij}}^{obs}(1,0)-\sum_{\left( i,j \right)\in T_{00}} {{{{I(M}_{i}=m)w}_{mi}^{*}w}_{i}}^{\left( m00 \right)}Y_{\mathrm{ij}}^{obs}\left( 0,0 \right).$$

This estimator is unbiased in a two-stage randomized design with a single allocation strategy and a control group (e.g., no agents assigned to PrEP) (39, 50). The estimators of the risk ratio of the spillover effect is defined analogously and can be estimated similarly to the risk difference.

We also consider stabilized versions of the weights and proposed Hajek-type estimators, which may provide more efficient estimators in a simulation setting and are a modest extension of existing estimators. We extended an expression for the spillover effect estimator and follow the proposed stabilized estimators described in (39). Let $\theta_{i}^{(10)}=\frac{1}{Pr\left( C_{i}=1 \right)Pr(A_{ij}=0)}$ and $\theta_{i}^{(00)}=\frac{1}{Pr(C_{i}=0)}$, then define:

$$\theta_{+}^{(10)}= \sum_{i} I\left( C_{i}=1 \right)\sum_{j} I\left( A_{ij}=0 \right)\theta_{i}^{(10)}$$

$$\theta_{+}^{(00)}=\sum_{i} I\left( C_{i}=0 \right)\sum_{j} I(A_{ij}=0)\theta_{i}^{(00)}$$

Let $I$ be the total number of components, $I_{1}$ be the number of intervention components, and $I_{0}$ be the number of control components. Let $n^{+}=\sum_{i} n_{i}$, $n_{00}^{+}= \sum_{i} I\left( C_{i}=0 \right)\sum_{j} I(A_{ij}=0)$ and $n_{10}^{+}= \sum_{i} I\left( C_{i}=1 \right)\sum_{j} I(A_{ij}=0)$. Then, $\theta_{i}^{(00)}=\frac{I}{I_{0}}$ and so $\theta_{+}^{(00)}=\frac{I}{I_{0}}n_{00}^{+}$. Also, $\theta_{i}^{(10)}=\frac{I}{I_{1}}\frac{1}{Pr(A_{ij}=0)}$ and so $\theta_{+}^{(10)}=n_{10}^{+}\frac{I}{I_{1}}\frac{1}{Pr(A_{ij}=0)}$. Then, with a control group with no agents randomized to the intervention,

$$E\left( \theta_{+}^{\left( 00 \right)} \right)=\frac{I}{I_{0}}\frac{I_{0}}{I}\sum_{i} n_{i}=n^{+}$$

$$E\left( \theta_{+}^{\left( 10 \right)} \right)=\frac{I}{I_{1}}\frac{I_{1}}{I}\sum_{i} \sum_{j} \frac{\Pr\left( A_{ij}=0 \right)}{Pr(A_{ij}=0)}=\sum_{i} n_{i}=n^{+}$$

Their proposed spillover effect estimator with stabilized weights is (33):

$$\hat{IE}=\frac{E\left( \theta_{+}^{\left( 10 \right)} \right)}{\theta_{+}^{\left( 10 \right)}}\sum_{i} \sum_{j} I\left( C_{i}=1 \right)I\left( A_{ij}=0 \right)\theta_{i}^{\left( 10 \right)}w_{i}^{*}Y_{ij}\left( 1,0 \right)- \frac{E\left( \theta_{+}^{\left( 00 \right)} \right)}{\theta_{+}^{\left( 00 \right)}}\sum_{i} \sum_{j} I\left( C_{i}=0 \right)\theta_{i}^{\left( 00 \right)}w_{i}^{*}Y_{ij}\left( 0,0 \right)$$

$=\frac{n^{+}}{n_{10}^{+}\frac{I}{I_{1}} \frac{1}{Pr(A_{ij}=0)}}\sum_{i} \sum_{j} I\left( C_{i}=1 \right)I\left( A_{ij}=0 \right)\theta_{i}^{\left( 10 \right)}w_{i}^{*}Y_{ij}\left( 1,0 \right)- \frac{n^{+}}{n_{00}^{+}\frac{I}{I_{0}}}\sum_{i} \sum_{j} I\left( C_{i}=0 \right)\theta_{i}^{\left( 00 \right)}w_{i}^{*}Y_{ij}\left( 0,0 \right)$.

When considering effect modification, we can estimate effects separately among the stratum determined by levels of $M$. Let $I_{m}$ be the total number of components with $M_{i}=m$, $I_{1m}$ be the number of intervention components with $M_{i}=m$, and $I_{0m}$ be the number of control components with $M_{i}=m$. Recall $n_{m}^{+}=\sum_{i} n_{i}I(M_{i}=m)$, and let $n_{m00}^{+}= \sum_{i} I(M_{i}=m)I\left( C_{i}=0 \right)\sum_{j} I(A_{ij}=0)$ and $n_{m10}^{+}= \sum_{i} I(M_{i}=m)I\left( C_{i}=1 \right)\sum_{j} I(A_{ij}=0)$. Then, $\theta_{mi}^{(m00)}=\frac{I_{m}}{I_{0m}}$ and so $\theta_{m+}^{(m00)}=\frac{I_{m}}{I_{0m}}n_{m00}^{+}$. Also, $\theta_{mi}^{(m10)}=\frac{I_{m}}{I_{1m}}\frac{1}{Pr(A_{ij}=0)}$ and so $\theta_{m+}^{(m10)}=n_{m10}^{+}\frac{I_{m}}{I_{1m}}\frac{1}{Pr(A_{ij}=0)}$. Then, the stabilized estimator of the spillover effect among components with $M_{i}=m$is:

$\hat{IE}_{m}=\frac{n_{m}^{+}}{n_{m10}^{+}\frac{I_{m}}{I_{1m}} \frac{1}{Pr(A_{ij}=0)}}\sum_{i} \sum_{j} I\left( M_{i}=m \right)I\left( C_{i}=1 \right)I\left( A_{ij}=0 \right)\theta_{i}^{\left( m10 \right)}w_{mi}^{*}Y_{ij}\left( 1,0 \right)- \frac{\sum_{i} \sum_{j} Pr(A_{ij}=0)}{n_{m00}^{+}\frac{I_{m}}{I_{0m}}}\sum_{i} \sum_{j} I\left( M_{i}=m \right)I\left( C_{i}=0 \right)\theta_{i}^{\left( m00 \right)}w_{mi}^{*}Y_{ij}\left( 0,0 \right)$.

**Supplementary Appendix 13: Network Structure of Components**

The network features considered were the bridging potential and density. The *network size* is defined as the number of nodes (i.e., agents) in the sexual network (51). An undirected *graph* $G = (V, E)$ is a mathematical structure consisting of a set $V$ of *vertices* or nodes (agents in our setting) and a set $E$ of *edges* or *links* (i.e., sexual partnerships), where elements of $E$are unordered pairs $\{u,v\}$ of distinct vertices $u,v \in V$.

A component’s *density* is defined as the proportion of observed connections in a component among the maximum number of possible connections in a component of the same size (51). The density $\rho_{i}$ is defined as the fraction of those edges that are actually present for component $i$:

$$\rho_{i}=\frac{2l_{i}}{n_{i}(n_{i}-1)},$$

where $n_{i}$ is the number of agents in the network component and $l_{i}$ is the number of edges (or connections) in the component. A sexual network component that is both large and dense is more likely to have agents who engage in sexual partnerships within the network component. This is particularly problematic when a pair of agents are HIV serodiscordant.

Bridging potential (also known as effective size) measures the redundancy in an individual agent’s partnerships by examining the connections between their partners, providing a measure of centrality of an individual agent where they could act as a mediator between two or more closely connected groups of agents (52). For our study with unweighted and undirected graphs with one type of node, a simplified formula to compute bridging potential for agent $u_{ij}$:

$$e\left( u_{ij} \right)=n_{ij}-\frac{2t_{ij}}{n_{ij}},$$

where $t_{ij}$is the number of edges (not including ties to agent $u_{ij}$) and $n_{ij}=n_{i}-1$ is the number of agents (excluding $u_{ij}$) in component $i$. Bridging potential can be used to identify critical agents for interrupting HIV transmission chains in a network component. Agents with high bridging potential can act as gatekeepers in the network (51) and, in the context of HIV, are agents who divide relatively isolated groups of other agents. If these agents remain uninfected, for example, by adhering to a PrEP regimen, they would limit or slow the spread of infection in the population (main paper Figure 1). Intervening on the HIV-negative agent with PrEP with no bridging potential would only protect that agent against HIV acquisition; however, intervening on the HIV-negative agent with PrEP with high bridging potential would protect that agent and the other HIV-negative agents in the component (53).

**Supplementary Table S1.** Summary of key model parameters in an agent-based model simulating a two-stage randomized trial overall and in a population of AAMSM and WMSM in Atlanta metropolitan area, 2015-2017

| **Domain** | **Overall** | **AAMSM** | **WMSM** | **Source** |
| --- | --- | --- | --- | --- |
| **Demographic Characteristics** |  |  |  |  |
| Population size (*n*) | 17,440 | 6,784 | 10,656 | (54) |
| Age distribution (%) |  |  |  | (54) |
| 18 to 24 years |  | 36.9 | 28.7 |  |
| 25 to 34 years |  | 46.3 | 53.6 |  |
| 35 to 49 years |  | 16.8 | 17.7 |  |
| Drug use |  | 30.0 | 48.5 | (11, 12) |
| **Sexual Behaviors** |  |  |  |  |
| Number of sex partners per year |  |  |  | (16) |
| Median (Interquartile Range) |  | 5 (3-10) | 7 (4-15) |  |
| Probability of condom use (%) |  | 68.8 | 52.8 | (21) |
| Probability of HIV transmission (per act) (%) |  |  |  |  |
| Condomless insertive anal intercourse | 0.11 |  |  | (23) |
| Condomless receptive anal intercourse | 1.38 |  |  | (23) |
| Sexual Role |  |  |  |  |
| Insertive only (%) |  | 24.2 | 22.8 | (14) |
| Receptive only (%) |  | 32.1 | 22.8 | (14) |
| Versatile (%) |  | 43.7 | 54.4 | (14) |
| **Pre-Exposure Prophylaxis Use (HIV- agents)** |  |  |  |  |
| Retention in clinical care, 24 months (%) | 100.0 |  |  | (29) |
| Optimal adherence (>4 pills per week) (%) |  | 91.1 | 56.8 | (55) |
| Reduction in risk of per-act HIV acquisition (%) | |  |  | (24) |
| Optimal adherence | 96.0 |  |  |  |
| Suboptimal adherence | 76.0 |  |  |  |
| **HIV and Cascade of Care** |  |  |  |  |
| HIV prevalence (% of all MSM) |  | 43.4 | 13.2 | (11) |
| HIV diagnosed (% of HIV+) |  | 65.5 | 81.8 | (25) |
| ART utilization (% of HIV+) |  | 43.1 | 48.8 | (25) |
| Virologically suppressed (% of HIV+) |  | 35.2 | 43.2 | (25) |
| AIDS (% of HIV+) |  | 51.7 | 53.1 |  |
| **Assortative Mixing** |  |  |  |  |
| WMSM with WMSM | 72.2 |  |  | (15) |
| AAMSM with AAMSM | 76.5 |  |  | (15) |
| Drug using agents with  drug using agents | 20.0 |  |  |  |

Abbreviation: men who have sex with men (MSM); African American/Black MSM (AAMSM); White MSM (WMSM).

**Supplementary Table S2.** Cumulative incidence of HIV over two years of follow-up after two-stage randomization stratified by four modifiers among agents within PrEP intervention (30% coverage) and control components in an agent-based model representing among men who have sex with men, Atlanta metropolitan area, Georgia, 2015-2017 (n = 3,911)

|  | Intervention Components | | | | | | Control Components | | |
| --- | --- | --- | --- | --- | --- | --- | --- | --- | --- |
|  | Agents on PrEP | | | Agents Not on PrEP | | | Agents Not on PrEP | | |
| Effect Modifiers | Total Agents | HIV+ | Cumulative Incidence | Total Agents | HIV+ | Cumulative Incidence | Total Agents | HIV+ | Cumulative Incidence |
| Overall | 386.3 | 4.7 | 0.01 | 900.5 | 84.5 | 0.09 | 1287.4 | 124.1 | 0.10 |
| Drug use |  |  |  |  |  |  |  |  |  |
| Among $M = 0$ | 208.9 | 2.9 | 0.01 | 487.1 | 50.4 | 0.10 | 695.3 | 73.8 | 0.11 |
| Among $M = 1$ | 177.4 | 1.8 | 0.01 | 413.4 | 34.1 | 0.08 | 592.1 | 50.4 | 0.09 |
| HIV Prevalence |  |  |  |  |  |  |  |  |  |
| Among $M = 0$ | 297.9 | 2.8 | 0.01 | 694.2 | 52.3 | 0.08 | 992.7 | 77.2 | 0.08 |
| Among $M = 1$ | 88.5 | 2.0 | 0.02 | 206.3 | 32.2 | 0.16 | 294.8 | 46.9 | 0.16 |
| Bridging Potential |  |  |  |  |  |  |  |  |  |
| Among $M = 0$ | 149.9 | 2.1 | 0.01 | 349.0 | 39.1 | 0.11 | 497.4 | 56.8 | 0.11 |
| Among $M = 1$ | 236.4 | 2.6 | 0.01 | 551.5 | 45.4 | 0.08 | 790.0 | 67.3 | 0.09 |
| Density |  |  |  |  |  |  |  |  |  |
| Among $M = 0$ | 305.0 | 3.5 | 0.01 | 712.5 | 61.9 | 0.09 | 1018.5 | 91.5 | 0.09 |
| Among $M = 1$ | 81.3 | 1.2 | 0.02 | 188.0 | 22.6 | 0.12 | 268.9 | 32.6 | 0.12 |

**Supplementary Table S3.** Estimated spillover effects of PrEP on cumulative incidence of HIV over two years of follow-up stratified by modifier *drug use* (M=0 vs. M=1) after two-stage randomization among agents within PrEP intervention (30% and 70% coverage) and control components in an agent-based model representing men who have sex with men, Atlanta metropolitan area, Georgia, 2015-2017 (n = 3,911 for 30% coverage; n = 3,947 for 70% coverage)^1^

|  | **Unstabilized** | | **Stabilized** | |
| --- | --- | --- | --- | --- |
| **Component Weighted** | RD (95% SI) | RR (95% SI) | RD (95% SI) | RR (95% SI) |
| M=0 |  |  |  |  |
| 30% Coverage | -0.03 (-0.09, 0.04) | 0.86 (0.52, 1.3) | -0.03 (-0.09, 0.04) | 0.86 (0.52, 1.3) |
| 70% Coverage | -0.09 (-0.14, -0.03) | 0.47 (0.24, 0.77) | -0.08 (-0.14, -0.03) | 0.47 (0.24, 0.78) |
| M=1 |  |  |  |  |
| 30% Coverage | -0.02 (-0.10, 0.06) | 0.90 (0.43, 1.6) | -0.02 (-0.10, 0.06) | 0.90 (0.43, 1.6) |
| 70% Coverage | -0.07 (-0.14, 0.00) | 0.53 (0.20, 1.0) | -0.07 (-0.14, 0.00) | 0.53 (0.20, 1.0) |
| **Individual Weighted** | RD (95% SI) | RR (95% SI) | RD (95% SI) | RR (95% SI) |
| M=0 |  |  |  |  |
| 30% Coverage | -0.02 (-0.07, 0.03) | 0.87 (0.54, 1.3) | -0.02 (-0.07, 0.03) | 0.87 (0.54, 1.3) |
| 70% Coverage | -0.07 (-0.11, -0.02) | 0.50 (0.27, 0.78) | -0.06 (-0.11, -0.02) | 0.50 (0.27, 0.78) |
| M=1 |  |  |  |  |
| 30% Coverage | -0.01 (-0.07, 0.04) | 0.91 (0.49, 1.5) | -0.01 (-0.07, 0.04) | 0.91 (0.49, 1.5) |
| 70% Coverage | -0.05 (-0.09, 0.00) | 0.57 (0.24, 1.0) | -0.05 (-0.09, 0.00) | 0.57 (0.25, 1.0) |

^1^ RD = Risk Difference; RR = Risk Ratio; SI = Simulation interval

**Supplementary Table S4.** Estimated spillover effects of PrEP on cumulative incidence of HIV over two years of follow-up stratified by modifier *HIV prevalence* (M=0 vs. M=1) after two-stage randomization among agents within PrEP intervention (30% and 70% coverage) and control components with 95% simulation intervals (SI) in an agent-based model representing men who have sex with men, Atlanta metropolitan area, Georgia, 2015-2017 (n = 3,911 for 30% coverage; n = 3,947 for 70% coverage)^1^

|  | Unstabilized | | Stabilized | |
| --- | --- | --- | --- | --- |
| **Component Weighted** | RD (95% SI) | RR (95% SI) | RD (95% SI) | RR (95% SI) |
| M=0 |  |  |  |  |
| 30% Coverage | -0.01 (-0.07, 0.04) | 0.92 (0.54, 1.5) | -0.01 (-0.07, 0.04) | 0.92 (0.54, 1.5) |
| 70% Coverage | -0.05 (-0.10, -0.01) | 0.56 (0.26, 0.91) | -0.05 (-0.10, -0.01) | 0.56 (0.26, 0.91) |
| M=1 |  |  |  |  |
| 30% Coverage | -0.05 (-0.16, 0.06) | 0.81 (0.43, 1.4) | -0.05 (-0.16, 0.06) | 0.81 (0.42, 1.4) |
| 70% Coverage | -0.13 (-0.22, -0.04) | 0.41 (0.18, 0.76) | -0.13 (-0.22, -0.04) | 0.41 (0.18, 0.76) |
| **Individual Weighted** | RD (95% SI) | RR (95% SI) | RD (95% SI) | RR (95% SI) |
| M=0 |  |  |  |  |
| 30% Coverage | -0.01 (-0.04, 0.03) | 0.93 (0.58, 1.4) | -0.01 (-0.04, 0.03) | 0.93 (0.59, 1.4) |
| 70% Coverage | -0.04 (-0.07, -0.01) | 0.60 (0.31, 0.94) | -0.04 (-0.07, -0.01) | 0.60 (0.31, 0.95) |
| M=1 |  |  |  |  |
| 30% Coverage | -0.03 (-0.12, 0.05) | 0.83 (0.47, 1.3) | -0.03 (-0.12, 0.05) | 0.84 (0.47, 1.3) |
| 70% Coverage | -0.10 (-0.17, -0.03) | 0.46 (0.21, 0.79) | -0.10 (-0.17, -0.03) | 0.46 (0.21, 0.80) |

^1^ RD = Risk Difference; RR = Risk Ratio; SI = Simulation interval

**Supplementary Table S5.** Estimated spillover effects of PrEP on cumulative incidence of HIV over two years of follow-up stratified by modifier *bridging potential* (M=0 vs. M=1) after two-stage randomization among agents within PrEP intervention (30% and 70% coverage) and control components with 95% simulation intervals (SI) in an agent-based model representing men who have sex with men, Atlanta metropolitan area, Georgia, 2015-2017 (n = 3,911 for 30% coverage; n = 3,947 for 70% coverage)^1^

|  | Unstabilized | | Stabilized | |
| --- | --- | --- | --- | --- |
| **Component Weighted** | RD (95% SI) | RR (95% SI) | RD (95% SI) | RR (95% SI) |
| M=0 |  |  |  |  |
| 30% Coverage | -0.02 (-0.08, 0.03) | 0.84 (0.50, 1.3) | -0.02 (-0.08, 0.03) | 0.84 (0.50, 1.3) |
| 70% Coverage | -0.08 (-0.12, -0.03) | 0.44 (0.23, 0.72) | -0.08 (-0.12, -0.03) | 0.44 (0.23, 0.72) |
| M=1 |  |  |  |  |
| 30% Coverage | -0.01 (-0.07, 0.05) | 0.96 (0.53, 1.6) | -0.01 (-0.06, 0.05) | 0.96 (0.53, 1.6) |
| 70% Coverage | -0.04 (-0.09, 0.02) | 0.68 (0.31, 1.2) | -0.04 (-0.09, 0.02) | 0.68 (0.31, 1.2) |
| **Individual Weighted** | RD (95% SI) | RR (95% SI) | RD (95% SI) | RR (95% SI) |
| M=0 |  |  |  |  |
| 30% Coverage | -0.02 (-0.08, 0.03) | 0.85 (0.50, 1.3) | -0.02 (-0.08, 0.03) | 0.85 (0.50, 1.3) |
| 70% Coverage | -0.07 (-0.12, -0.03) | 0.44 (0.23, 0.71) | -0.07 (-0.12, -0.03) | 0.44 (0.23, 0.71) |
| M=1 |  |  |  |  |
| 30% Coverage | -0.01 (-0.05, 0.04) | 0.96 (0.56, 1.50) | -0.01 (0.05, 0.04) | 0.96 (0.56, 1.5) |
| 70% Coverage | -0.03 (-0.08, 0.02) | 0.70 (0.34, 1.21) | -0.03 (-0.07, 0.02) | 0.70 (0.34, 1.2) |

^1^ RD = Risk Difference; RR = Risk Ratio; SI = Simulation interval

**Supplementary Table S6.** Estimated spillover effects of PrEP on cumulative incidence of HIV over two years of follow-up stratified by modifier *density* (M=0 vs. M=1) after two-stage randomization among agents within PrEP intervention (30% and 70% coverage) and control components with 95% simulation intervals (SI) in an agent-based model representing men who have sex with men, Atlanta metropolitan area, Georgia, 2015-2017 (n = 3,911 for 30% coverage; n = 3,947 for 70% coverage)^1^

|  | Unstabilized | | Stabilized | |
| --- | --- | --- | --- | --- |
| **Component Weighted** | RD (95% SI) | RR (95% SI) | RD (95% SI) | RR (95% SI) |
| M=0 |  |  |  |  |
| 30% Coverage | -0.01 (-0.07, 0.04) | 0.91 (0.54, 1.4) | -0.01 (-0.07, 0.04) | 0.92 (0.54, 1.4) |
| 70% Coverage | -0.06 (-0.10, -0.01) | 0.58 (0.30, 0.94) | -0.06 (-0.10, -0.01) | 0.58 (0.30, 0.94) |
| M=1 |  |  |  |  |
| 30% Coverage | -0.03 (-0.09, 0.04) | 0.83 (0.45, 1.4) | -0.03 (-0.09, 0.04) | 0.84 (0.45, 1.4) |
| 70% Coverage | -0.08 (-0.14, -0.02) | 0.42 (0.17, 0.79) | -0.08 (-0.14, -0.02) | 0.42 (0.17, 0.79) |
| **Individual Weighted** | RD (95% SI) | RR (95% SI) | RD (95% SI) | RR (95% SI) |
| M=0 |  |  |  |  |
| 30% Coverage | -0.01 (-0.05, 0.03) | 0.92 (0.59, 1.3) | -0.01 (-0.05, 0.03) | 0.92 (0.59, 1.3) |
| 70% Coverage | -0.04 (-0.08, -0.01) | 0.61 (0.34, 0.93) | -0.04 (-0.08, -0.01) | 0.62 (0.34, 0.93) |
| M=1 |  |  |  |  |
| 30% Coverage | -0.03 (-0.09, 0.04) | 0.83 (0.45, 1.4) | -0.03 (-0.09, 0.04) | 0.84 (0.45, 1.4) |
| 70% Coverage | -0.08 (-0.14, -0.02) | 0.42 (0.17, 0.79) | -0.08 (-0.14, -0.02) | 0.42 (0.17, 0.79) |

^1^ RD = Risk Difference; RR = Risk Ratio; SI = Simulation interval

**Supplementary Table S7**. Characteristics of components at the time of enrollment into the simulated two-stage randomized trial with 70% PrEP coverage in the intervention group in an agent-based model representing men who have sex with men in the Atlanta metropolitan area, Georgia, 2015-2017, *excluding components with only one HIV-negative agent*^1^

|  |  | **Component Assignment** | |
| --- | --- | --- | --- |
| **Characteristics** | **Summary Measure** | **Intervention**  **(n=1801.04)** | **Control**  **(n=1795.09)** |
| Number of components |  | 347.0 | 346.1 |
| Average component size | Mean (SD) | 5.2 (3.3) | 5.2 (3.3) |
|  | Median (IQR) | 4.0 (3.0) | 4.0 (3.0) |
| HIV prevalence |  | 30.8% | 30.8% |
| African American race |  | 42.5% | 42.5% |
| Any drug use |  | 35.7% | 35.7% |
| Bridging potential | Mean (SD) | 1.5 (0.17) | 1.5 (0.17) |
|  | Median (IQR) | 1.5 (0.33) | 1.5 (0.33) |
| Density | Mean (SD) | 0.47 (0.17) | 0.47 (0.17) |
|  | Median (IQR) | 0.50 (0.33) | 0.50 (0.33) |

^1^ Results above are from 1,000 iterations of the agent-based model. SD = standard deviation; IQR = interquartile range

**Supplementary Table S8.** Cumulative incidence of HIV over two years of follow-up after two-stage randomization stratified by four modifiers among agents within PrEP intervention (70% coverage) and control components in an agent-based model representing among men who have sex with men, Atlanta metropolitan area, Georgia, 2015-2017 (n = 3,596), *excluding components with only one HIV-negative agent*

|  | Intervention Components | | | | | | Control Components | | |
| --- | --- | --- | --- | --- | --- | --- | --- | --- | --- |
|  | Agents on PrEP | | | Agents Not on PrEP | | | Agents Not on PrEP | | |
| Effect Modifiers | Total Agents | HIV+ | Cumulative Incidence | Total Agents | HIV+ | Cumulative Incidence | Total Agents | HIV+ | Cumulative Incidence |
| Overall | 872.0 | 9.3 | 0.01 | 373.4 | 32.0 | 0.09 | 1252.1 | 113.8 | 0.09 |
| Drug use |  |  |  |  |  |  |  |  |  |
| Among $M = 0$ | 462.6 | 5.5 | 0.01 | 198.1 | 18.5 | 0.09 | 659.4 | 65.7 | 0.10 |
| Among $M = 1$ | 409.4 | 3.7 | 0.01 | 175.3 | 13.5 | 0.08 | 582.7 | 48.1 | 0.08 |
| HIV Prevalence |  |  |  |  |  |  |  |  |  |
| Among $M = 0$ | 700.1 | 6.0 | 0.01 | 300.0 | 21.6 | 0.07 | 998.0 | 77.5 | 0.08 |
| Among $M = 1$ | 171.9 | 3.2 | 0.02 | 73.4 | 10.4 | 0.14 | 244.0 | 36.3 | 0.15 |
| Bridging Potential |  |  |  |  |  |  |  |  |  |
| Among $M = 0$ | 316.1 | 3.8 | 0.01 | 134.8 | 13.2 | 0.10 | 449.5 | 46.5 | 0.10 |
| Among $M = 1$ | 555.9 | 5.5 | 0.01 | 238.6 | 18.8 | 0.08 | 792.6 | 67.4 | 0.09 |
| Density |  |  |  |  |  |  |  |  |  |
| Among $M = 0$ | 711.8 | 7.2 | 0.01 | 304.8 | 25.1 | 0.08 | 1014.1 | 89.7 | 0.09 |
| Among $M = 1$ | 160.2 | 2.0 | 0.01 | 68.6 | 6.9 | 0.10 | 227.9 | 24.1 | 0.11 |

**Supplementary Table S9.** Estimated spillover effects of PrEP on cumulative incidence of HIV over two years of follow-up stratified by modifier *drug use* (M=0 vs. M=1) after two-stage randomization among agents within PrEP intervention (70% coverage) and control components in an agent-based model representing men who have sex with men, Atlanta metropolitan area, Georgia, 2015-2017 (n = 3,596), *excluding components with only one HIV-negative agent*^1^

|  | **Unstabilized** | | **Stabilized** | |
| --- | --- | --- | --- | --- |
| **Component Weighted** | RD (95% SI) | RR (95% SI) | RD (95% SI) | RR (95% SI) |
| M=0 |  |  |  |  |
| Drug use | -0.06 (-0.12, -0.01) | 0.55 (0.25, 0.93) | -0.06 (-0.12, -0.01) | 0.55 (0.25, 0.93) |
| HIV prevalence | -0.05 (-0.10, -0.01) | 0.56 (0.26, 0.91) | -0.05 (-0.10, -0.01) | 0.56 (0.26, 0.91) |
| Bridging potential | -0.05 (-0.10, -0.01) | 0.52 (0.24, 0.90) | -0.05 (-0.10, -0.01) | 0.52 (0.24, 0.90) |
| Density | -0.05 (-0.09, -0.00) | 0.62 (0.32, 0.99) | -0.05 (-0.09, -0.00) | 0.62 (0.32, 0.99) |
| M=1 |  |  |  |  |
| Drug use | -0.05 (-0.11, 0.01) | 0.60 (0.22, 1.1) | -0.05 (-0.11, 0.01) | 0.60 (0.22, 1.1) |
| HIV prevalence | -0.08 (-0.17, 0.02) | 0.58 (0.20, 1.2) | -0.08 (-0.17, 0.02) | 0.58 (0.20, 1.2) |
| Bridging potential | -0.04 (-0.09, 0.02) | 0.70 (0.31, 1.2) | -0.04 (-0.09, 0.02) | 0.70 (0.31, 1.2) |
| Density | -0.06 (-0.11, 0.00) | 0.50 (0.16, 1.1) | -0.06 (-0.11, 0.00) | 0.50 (0.16, 1.1) |
| **Individual Weighted** | RD (95% SI) | RR (95% SI) | RD (95% SI) | RR (95% SI) |
| M=0 |  |  |  |  |
| Drug use | -0.05 (-0.09, -0.01) | 0.58 (0.27, 0.95) | -0.05 (-0.09, -0.01) | 0.58 (0.27, 0.95) |
| HIV prevalence | -0.04 (-0.07, -0.01) | 0.60 (0.31, 0.95) | -0.04 (-0.07, -0.01) | 0.60 (0.31, 0.95) |
| Bridging potential | -0.05 (-0.10, -0.01) | 0.52 (0.24, 0.89) | -0.05 (-0.10, -0.01) | 0.52 (0.24, 0.89) |
| Density | -0.04 (-0.07, 0.00) | 0.65 (0.36, 1.01) | -0.04 (-0.07, 0.00) | 0.65 (0.36, 1.01) |
| M=1 |  |  |  |  |
| Drug use | -0.04 (-0.08, 0.01) | 0.64 (0.26, 1.1) | -0.04 (-0.08, 0.01) | 0.64 (0.26, 1.1) |
| HIV prevalence | -0.06 (-0.15, 0.01) | 0.60 (0.23, 1.1) | -0.06 (-0.15, 0.01) | 0.60 (0.23, 1.1) |
| Bridging potential | -0.03 (-0.07, 0.02) | 0.72 (0.34, 1.2) | -0.03 (-0.07, 0.02) | 0.72 (0.34, 1.2) |
| Density | -0.06 (-0.11, 0.00) | 0.50 (0.16, 1.1) | -0.06 (-0.11, 0.00) | 0.50 (0.16, 1.1) |

^1^ RD = Risk Difference; RR = Risk Ratio; SI = Simulation interval

**Supplementary Table S10.** Sensitivity analyses of PrEP adherence: HIV prevalence and cumulative incidence in a two-stage randomization with 70% coverage in the intervention group in an agent-based model representing among men who have sex with men, Atlanta metropolitan area, Georgia, 2015-2017, stratified by the effect modifier $M$ (drug use).

| **Scenario** | **HIV Prevalence** | | | **Cumulative Incidence** | | |
| --- | --- | --- | --- | --- | --- | --- |
|  | **Intervention**  **Agents on PrEP** | **Intervention Agents not on PrEP** | **Control Agents** | **Intervention**  **Agents on PrEP** | **Intervention Agents not on PrEP** | **Control Agents** |
| **Among Components with *M* = 1** | | | | | | |
| Main | 4.0 | 291.8 | 327.4 | 4.0 | 14.3 | 51.0 |
| *PrEP Adherence* |  |  |  |  |  |  |
| 80% (White MSM)  50% (Black MSM) | 4.6 | 291.2 | 329.8 | 4.6 | 14.3 | 51.4 |
| **Among Components with *M* = 0** | | | | | | |
| Main | 6.4 | 421.1 | 471.6 | 6.4 | 21.1 | 74.2 |
| *PrEP Adherence* |  |  |  |  |  |  |
| 80% (White MSM)  50% (Black MSM) | 7.5 | 422.4 | 478.0 | 7.5 | 21.4 | 74.9 |

**Supplementary Table S11.** Sensitivity analyses of PrEP adherence: HIV prevalence and cumulative incidence in a two-stage randomization with 70% coverage in the intervention group in an agent-based model representing among men who have sex with men, Atlanta metropolitan area, Georgia, 2015-2017, stratified by the effect modifier $M$ (HIV prevalence).

| **Scenario** | **HIV Prevalence** | | | **Cumulative Incidence** | | |  |
| --- | --- | --- | --- | --- | --- | --- | --- |
|  | **Intervention**  **Agents on PrEP** | **Intervention Agents not on PrEP** | **Control Agents** | **Intervention**  **Agents on PrEP** | **Intervention Agents not on PrEP** | **Control Agents** | |
| **Among Components with *M* = 1** | | | | | | |  |
| Main | 4.4 | 358.0 | 389.3 | 4.4 | 13.8 | 47.7 | |
| *PrEP Adherence* |  |  |  |  |  |  | |
| 80% (White MSM)  50% (Black MSM) | 5.1 | 358.4 | 395.0 | 5.1 | 14.2 | 48.5 | |
| **Among Components with *M* = 0** | | | | | | |  |
| Main | 6.0 | 354.9 | 409.8 | 6.0 | 21.6 | 77.5 | |
| *PrEP Adherence* |  |  |  |  |  |  | |
| 80% (White MSM)  50% (Black MSM) | 7.1 | 355.2 | 412.8 | 7.1 | 21.6 | 77.9 | |

**Supplementary Table S12.** Sensitivity analyses of PrEP adherence: HIV prevalence and cumulative incidence in a two-stage randomization with 70% coverage in the intervention group in an agent-based model representing among men who have sex with men, Atlanta metropolitan area, Georgia, 2015-2017, stratified by the effect modifier $M$ (bridging potential).

| **Scenario** | **HIV Prevalence** | | | **Cumulative Incidence** | | |
| --- | --- | --- | --- | --- | --- | --- |
|  | **Intervention**  **Agents on PrEP** | **Intervention Agents not on PrEP** | **Control Agents** | **Intervention**  **Agents on PrEP** | **Intervention Agents not on PrEP** | **Control Agents** |
| **Among Components with *M* = 1** | | | | | | |
| Main | 5.5 | 357.1 | 404.2 | 5.5 | 19.0 | 67.9 |
| *PrEP Adherence* |  |  |  |  |  |  |
| 80% (White MSM)  50% (Black MSM) | 6.5 | 356.3 | 408.9 | 6.5 | 19.1 | 68.4 |
| **Among Components with *M* = 0** | | | | | | |
| Main | 4.9 | 355.8 | 394.8 | 4.9 | 16.4 | 57.3 |
| *PrEP Adherence* |  |  |  |  |  |  |
| 80% (White MSM)  50% (Black MSM) | 5.7 | 357.3 | 398.9 | 5.7 | 16.7 | 57.9 |

**Supplementary Table S13.** Sensitivity analyses of PrEP adherence: HIV prevalence and cumulative incidence in a two-stage randomization with 70% coverage in the intervention group in an agent-based model representing among men who have sex with men, Atlanta metropolitan area, Georgia, 2015-2017, stratified by the effect modifier $M$ (density).

| **Scenario** | **HIV Prevalence** | | | **Cumulative Incidence** | | |
| --- | --- | --- | --- | --- | --- | --- |
|  | **Intervention**  **Agents on PrEP** | **Intervention Agents not on PrEP** | **Control Agents** | **Intervention**  **Agents on PrEP** | **Intervention Agents not on PrEP** | **Control Agents** |
| **Among Components with *M* = 1** | | | | | | |
| Main | 2.8 | 210.3 | 232.8 | 2.9 | 9.6 | 33.0 |
| *PrEP Adherence* |  |  |  |  |  |  |
| 80% (White MSM)  50% (Black MSM) | 3.4 | 211.2 | 235.1 | 3.4 | 9.7 | 33.4 |
| **Among Components with *M* = 0** | | | | | | |
| Main | 7.5 | 502.6 | 566.2 | 7.5 | 25.8 | 92.2 |
| *PrEP Adherence* |  |  |  |  |  |  |
| 80% (White MSM)  50% (Black MSM) | 8.8 | 502.4 | 572.7 | 8.8 | 26.1 | 92.9 |

**Supplementary Table S14.** Sensitivity analyses of PrEP discontinuation: HIV prevalence and cumulative incidence in a two-stage randomization with 70% coverage in the intervention group in an agent-based model representing among men who have sex with men, Atlanta metropolitan area, Georgia, 2015-2017, stratified by the effect modifier $M$ (drug use).

| **Scenario** | **HIV Prevalence** | | | **Cumulative Incidence** | | |
| --- | --- | --- | --- | --- | --- | --- |
|  | **Intervention**  **Agents on PrEP** | **Intervention Agents not on PrEP** | **Control Agents** | **Intervention**  **Agents on PrEP** | **Intervention Agents not on PrEP** | **Control Agents** |
| **Among Components with *M* = 1** | | | | | | |
| Main | 4.0 | 291.8 | 327.4 | 4.0 | 14.3 | 51.0 |
| *PrEP Discontinuation* |  |  |  |  |  |  |
| 10% | 24.6 | 287.5 | 323.9 | 24.6 | 13.4 | 50.4 |
| **Among Components with *M* = 0** | | | | | | |
| Main | 6.4 | 421.1 | 471.6 | 6.4 | 21.1 | 74.2 |
| *PrEP Discontinuation* |  |  |  |  |  |  |
| 10% | 36.0 | 414.0 | 467.6 | 36.0 | 21.4 | 73.5 |

**Supplementary Table S15.** Sensitivity analyses of PrEP discontinuation: HIV prevalence and cumulative incidence in a two-stage randomization with 70% coverage in the intervention group in an agent-based model representing among men who have sex with men, Atlanta metropolitan area, Georgia, 2015-2017, stratified by the effect modifier $M$ (HIV prevalence).

| **Scenario** | **HIV Prevalence** | | | **Cumulative Incidence** | | |
| --- | --- | --- | --- | --- | --- | --- |
|  | **Intervention**  **Agents on PrEP** | **Intervention Agents not on PrEP** | **Control Agents** | **Intervention**  **Agents on PrEP** | **Intervention Agents not on PrEP** | **Control Agents** |
| **Among Components with *M* = 1** | | | | | | |
| Main | 4.4 | 358.0 | 389.3 | 4.4 | 13.8 | 47.7 |
| *PrEP Discontinuation* |  |  |  |  |  |  |
| 10% | 23.5 | 351.1 | 385.4 | 23.5 | 13.8 | 47.2 |
| **Among Components with *M* = 0** | | | | | | |
| Main | 6.0 | 354.9 | 409.8 | 6.0 | 21.6 | 77.5 |
| *PrEP Discontinuation* |  |  |  |  |  |  |
| 10% | 37.1 | 350.4 | 406.1 | 37.1 | 22.1 | 76.8 |

**Supplementary Table S16.** Sensitivity analyses of PrEP discontinuation: HIV prevalence and cumulative incidence in a two-stage randomization with 70% coverage in the intervention group in an agent-based model representing among men who have sex with men, Atlanta metropolitan area, Georgia, 2015-2017, stratified by the effect modifier $M$ (bridging potential).

| **Scenario** | **HIV Prevalence** | | | **Cumulative Incidence** | | |
| --- | --- | --- | --- | --- | --- | --- |
|  | **Intervention**  **Agents on PrEP** | **Intervention Agents not on PrEP** | **Control Agents** | **Intervention**  **Agents on PrEP** | **Intervention Agents not on PrEP** | **Control Agents** |
| **Among Components with *M* = 1** | | | | | | |
| Main | 5.5 | 357.1 | 404.2 | 5.5 | 19.0 | 67.9 |
| *PrEP Discontinuation* |  |  |  |  |  |  |
| 10% | 32.7 | 350.6 | 400.1 | 32.7 | 19.3 | 67.3 |
| **Among Components with *M* = 0** | | | | | | |
| Main | 4.9 | 355.8 | 394.8 | 4.9 | 16.4 | 57.3 |
| *PrEP Discontinuation* |  |  |  |  |  |  |
| 10% | 27.9 | 350.8 | 391.4 | 27.9 | 16.6 | 56.7 |

**Supplementary Table S17.** Sensitivity analyses of PrEP discontinuation: HIV prevalence and cumulative incidence in a two-stage randomization with 70% coverage in the intervention group in an agent-based model representing among men who have sex with men, Atlanta metropolitan area, Georgia, 2015-2017, stratified by the effect modifier $M$ (density).

| **Scenario** | **HIV Prevalence** | | | **Cumulative Incidence** | | |
| --- | --- | --- | --- | --- | --- | --- |
|  | **Intervention**  **Agents on PrEP** | **Intervention Agents not on PrEP** | **Control Agents** | **Intervention**  **Agents on PrEP** | **Intervention Agents not on PrEP** | **Control Agents** |
| **Among Components with *M* = 1** | | | | | | |
| Main | 2.8 | 210.3 | 232.8 | 2.9 | 9.6 | 33.0 |
| *PrEP Discontinuation* |  |  |  |  |  |  |
| 10% | 16.1 | 208.1 | 230.0 | 16.1 | 9.7 | 32.5 |
| **Among Components with *M* = 0** | | | | | | |
| Main | 7.5 | 502.6 | 566.2 | 7.5 | 25.8 | 92.2 |
| *PrEP Discontinuation* |  |  |  |  |  |  |
| 10% | 44.5 | 493.4 | 561.5 | 44.5 | 26.2 | 91.5 |

**Supplementary Table S18.** Sensitivity analyses with lower PrEP adherence or PrEP discontinuation: Estimated spillover risk differences (RD) and risk ratios (RR) with 95% simulation intervals (SI) for HIV cumulative incidence in a two-stage randomization with 70% coverage in the intervention group in an agent-based model representing among men who have sex with men, Atlanta metropolitan area, Georgia, 2015-2017, stratified by the effect modifier $M$ (drug use).

| **Scenario** | RD (95% SI) | RR (95% SI) |
| --- | --- | --- |
| **Among Components with *M* = 1** | | |
| Main | -0.07 (-0.14, 0.00) | 0.53 (0.20, 1.0) |
| *PrEP Adherence* |  |  |
| 80% (White MSM)  50% (Black MSM) | -0.07 (-0.13, 0.00) | 0.53 (0.21, 1.0) |
| *PrEP Discontinuation* |  |  |
| 10% | -0.07 (-0.14, -0.001) | 0.51 (0.18, 0.99) |
| **Among Components with *M* = 0** | | |
| Main | -0.08 (-0.14, -0.13) | 0.47 (0.24, 0.78) |
| *PrEP Adherence* |  |  |
| 80% (White MSM)  50% (Black MSM) | -0.08 (-0.14, -0.03) | 0.48 (0.24, 0.80) |
| *PrEP Discontinuation* |  |  |
| 10% | -0.09 (-0.14, -0.03) | 0.46 (0.22, 0.79) |

**Supplementary Table S19.** Sensitivity analyses with lower PrEP adherence or PrEP discontinuation: Estimated spillover risk differences (RD) and risk ratios (RR) with 95% simulation intervals (SI) for HIV cumulative incidence in a two-stage randomization with 70% coverage in the intervention group in an agent-based model representing among men who have sex with men, Atlanta metropolitan area, Georgia, 2015-2017, stratified by the effect modifier $M$ (HIV prevalence).

| **Scenario** | RD (95% SI) | RR (95% SI) |
| --- | --- | --- |
| **Among Components with *M* = 1** | | |
| Main | -0.13 (-0.22, -0.04) | 0.41 (0.18, 0.76) |
| *PrEP Adherence* |  |  |
| 80% (White MSM)  50% (Black MSM) | -0.13 (-0.22, -0.04) | 0.43 (0.18, 0.76) |
| *PrEP Discontinuation* |  |  |
| 10% | -0.13 (-0.22, -0.03) | 0.41 (0.16, 0.81) |
| **Among Components with *M* = 0** | | |
| Main | -0.05 (-0.10, -0.01) | 0.56 (0.26, 0.91) |
| *PrEP Adherence* |  |  |
| 80% (White MSM)  50% (Black MSM) | -0.05 (-0.10, 0.00) | 0.56 (0.25, 0.98) |
| *PrEP Discontinuation* |  |  |
| 10% | -0.05 (-0.10, -0.004) | 0.54 (0.26, 0.96) |

**Supplementary Table S20.** Sensitivity analyses with lower PrEP adherence or PrEP discontinuation: Estimated spillover risk differences (RD) and risk ratios (RR) with 95% simulation intervals (SI) for HIV cumulative incidence in a two-stage randomization with 70% coverage in the intervention group in an agent-based model representing among men who have sex with men, Atlanta metropolitan area, Georgia, 2015-2017, stratified by the effect modifier $M$ (bridging potential).

| **Scenario** | RD (95% SI) | RR (95% SI) |
| --- | --- | --- |
| **Among Components with *M* = 1** | | |
| Main | -0.04 (-0.09, 0.02) | 0.68 (0.31, 1.2) |
| *PrEP Adherence* |  |  |
| 80% (White MSM)  50% (Black MSM) | -0.04 (-0.10, 0.02) | 0.68 (0.31, 1.2) |
| *PrEP Discontinuation* |  |  |
| 10% | -0.05 (-0.11, 0.02) | 0.63 (0.26, 1.2) |
| **Among Components with *M* = 0** | | |
| Main | -0.08 (-0.12, -0.03) | 0.44 (0.23, 0.72) |
| *PrEP Adherence* |  |  |
| 80% (White MSM)  50% (Black MSM) | -0.07 (-0.12, -0.03) | 0.44 (0.23, 0.73) |
| *PrEP Discontinuation* |  |  |
| 10% | -0.08 (-0.12, -0.03) | 0.44 (0.21, 0.75) |

**Supplementary Table S21.** Sensitivity analyses with lower PrEP adherence or PrEP discontinuation: Estimated spillover risk differences (RD) and risk ratios (RR) with 95% simulation intervals (SI) for HIV cumulative incidence in a two-stage randomization with 70% coverage in the intervention group in an agent-based model representing among men who have sex with men, Atlanta metropolitan area, Georgia, 2015-2017, stratified by the effect modifier $M$ (density).

| **Scenario** | RD (95% SI) | RR (95% SI) |
| --- | --- | --- |
| **Among Components with *M* = 1** | | |
| Main | -0.08 (-0.14, -0.02) | 0.42 (0.17, 0.79) |
| *PrEP Adherence* |  |  |
| 80% (White MSM)  50% (Black MSM) | -0.08 (-0.13, -0.02) | 0.42 (0.16, 0.79) |
| *PrEP Discontinuation* |  |  |
| 10% | -0.08 (-0.14, -0.02) | 0.42 (0.15, 0.80) |
| **Among Components with *M* = 0** | | |
| Main | -0.06 (-0.10, -0.01) | 0.58 (0.30, 0.94) |
| *PrEP Adherence* |  |  |
| 80% (White MSM)  50% (Black MSM) | -0.06 (-0.10, -0.01) | 0.59 (0.32, 0.95) |
| *PrEP Discontinuation* |  |  |
| 10% | -0.06 (-0.11, -0.01) | 0.55 (0.29, 0.93) |

**Supplementary Figure S1.** Estimated spillover risk differences of PrEP on cumulative incidence of HIV by effect modifiers (M = 1 if prevalence above median vs. M = 0 at or below median) among HIV-negative agents within PrEP intervention (70% coverage) and control components in two-stage randomized designs of a PrEP intervention with 70% coverage in an agent-based model representing men who have sex with men, Atlanta metropolitan area, Georgia, 2015-2017. Lines within boxes, median values; box borders, interquartile ranges (75th and 25th percentiles); bars, 90th and 10th percentiles; points, outliers. Shaded shape represented the distribution of estimates and dashed lines represent the null value (n = 3,596), *excluding components with only one HIV-negative agent*

**
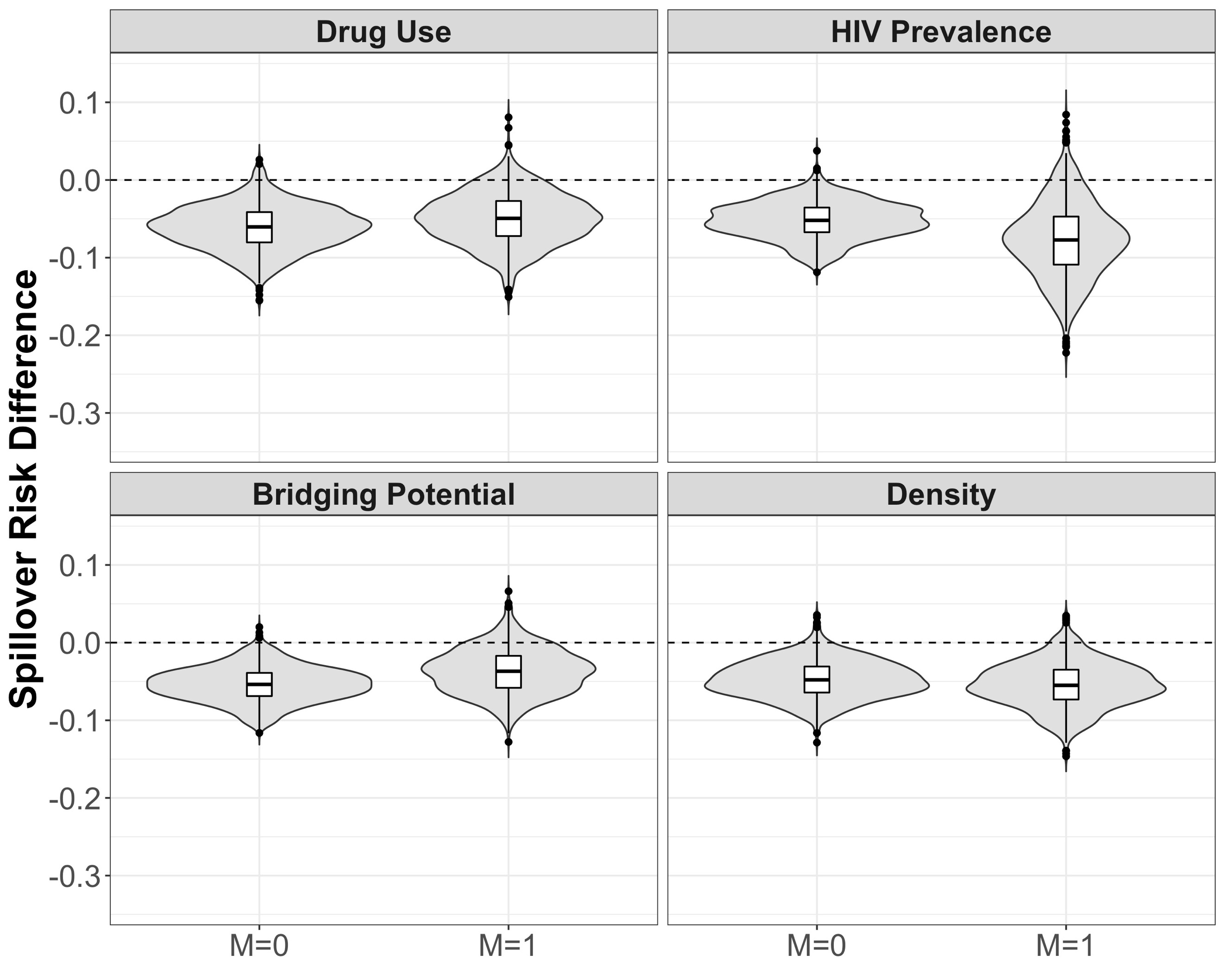
**

**Supplementary Figure S2.** Estimated spillover risk ratios of PrEP on cumulative incidence of HIV by effect modifiers (M = 1 if prevalence above median vs. M = 0 at or below median) among HIV-negative agents within PrEP intervention (70% coverage) and control components in two-stage randomized designs of a PrEP intervention with 70% coverage in an agent-based model representing men who have sex with men, Atlanta metropolitan area, Georgia, 2015-2017. Lines within boxes, median values; box borders, interquartile ranges (75th and 25th percentiles); bars, 90th and 10th percentiles; points, outliers. Shaded shape represented the distribution of estimates and dashed lines represent the null value (n = 3,596), *excluding components with only one HIV-negative agent*

**
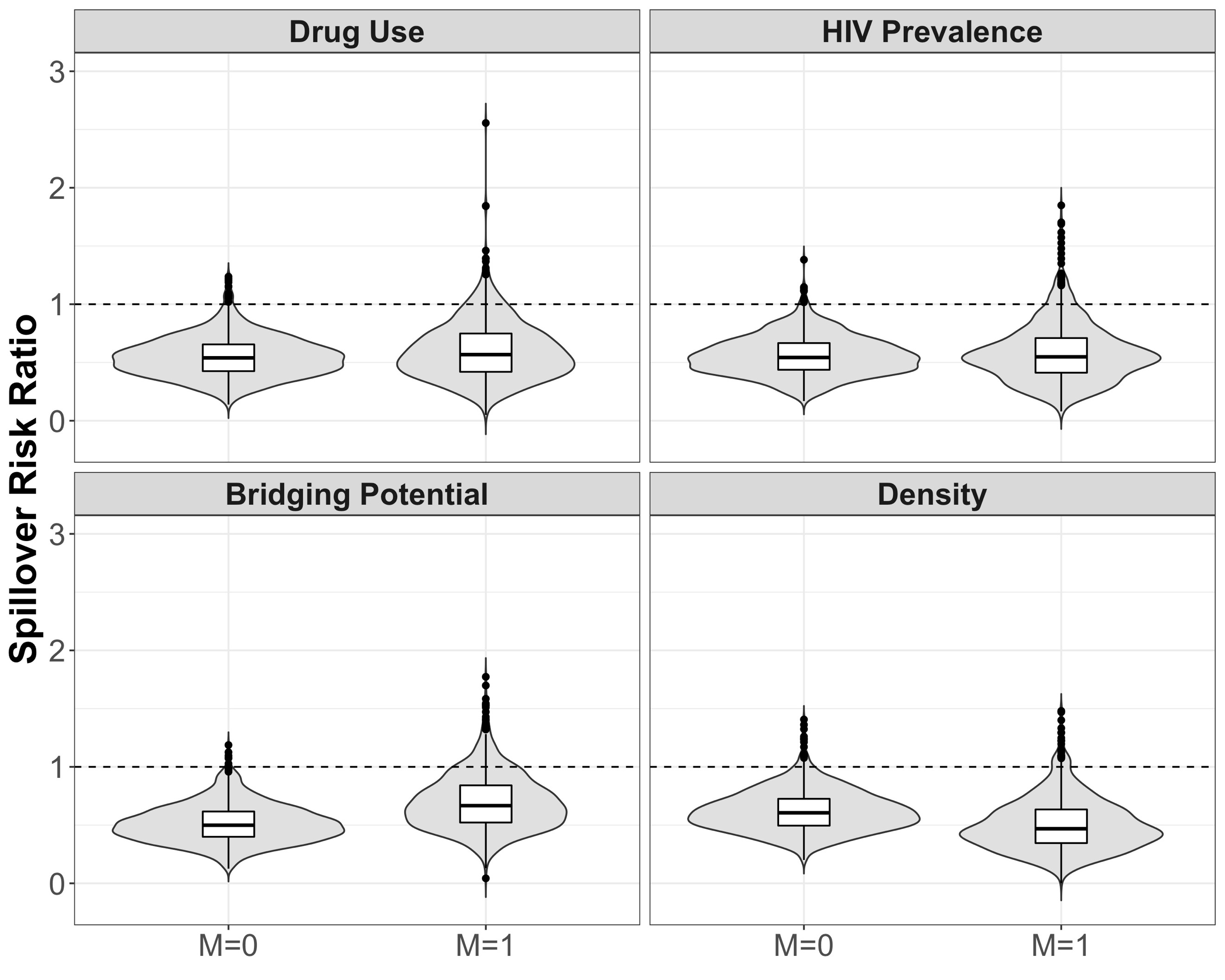
**

**Supplementary Figure S3.** Estimated risk difference spillover effects of PrEP on cumulative incidence of HIV by four modifiers (M = 1 if prevalence above median vs. M = 0 at or below median) in two-stage randomized designs of a pre-exposure prophylaxis (PrEP) intervention with 70% coverage in an agent-based model representing men who have sex with men, Atlanta metropolitan area, Georgia, 2015-2017 and *PrEP adherence set to 50% among Black MSM and 80% among White MSM***.** Lines within boxes, median values; box borders, interquartile ranges (75th and 25th percentiles); bars, 90th and 10th percentiles; points, outliers. Shaded shape represented the distribution of estimates and dashed lines represent the null value. (n = 3,967).

**
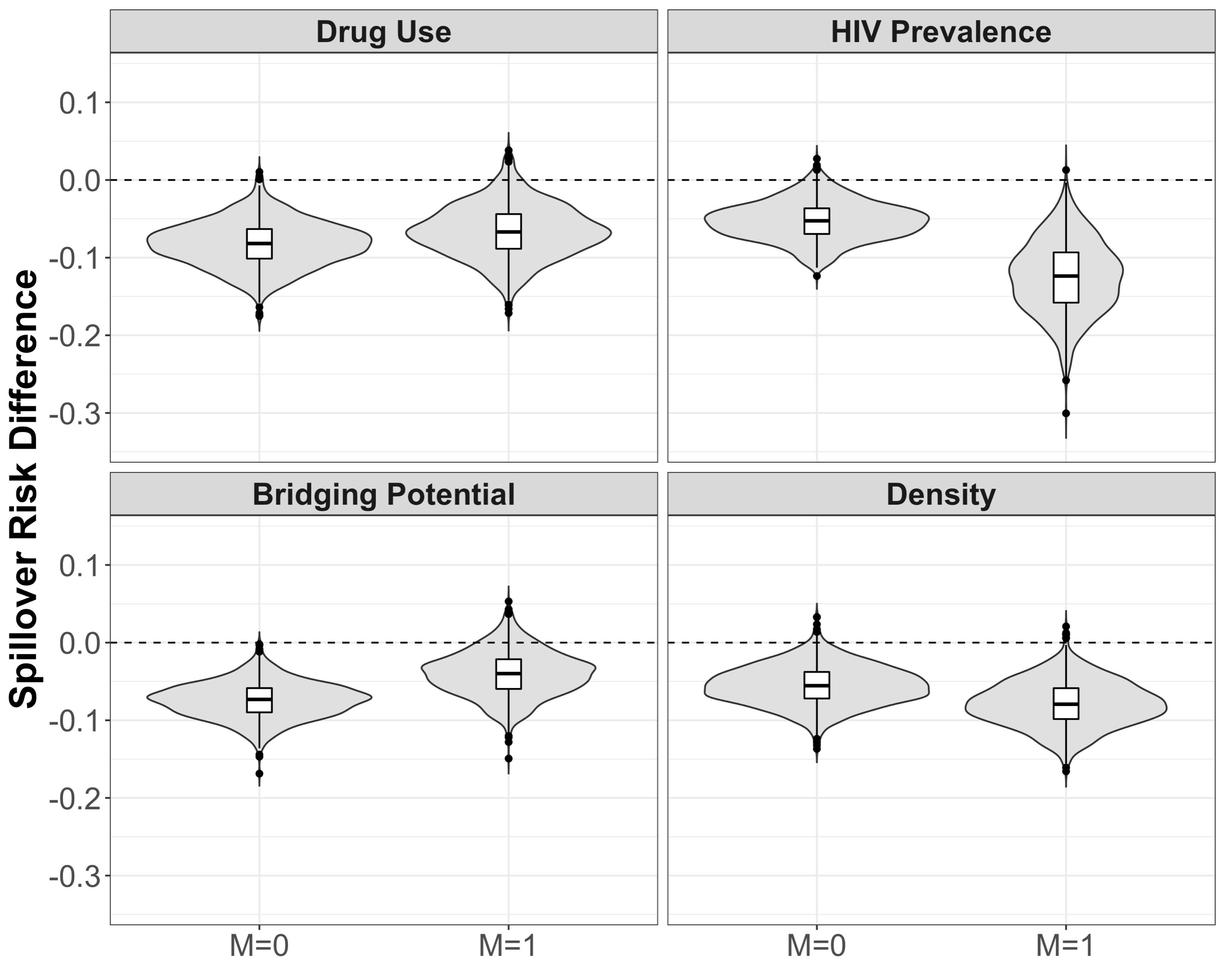
**

**Supplementary Figure S4.** Estimated risk ratio spillover effects of PrEP on cumulative incidence of HIV by four modifiers (M = 1 if prevalence above median vs. M = 0 at or below median) in two-stage randomized designs of a pre-exposure prophylaxis (PrEP) intervention with 70% coverage in an agent-based model representing men who have sex with men, Atlanta metropolitan area, Georgia, 2015-2017 and *PrEP adherence set to 50% among Black MSM and 80% among White MSM*. Lines within boxes, median values; box borders, interquartile ranges (75th and 25th percentiles); bars, 90th and 10th percentiles; points, outliers. Shaded shape represented the distribution of estimates and dashed lines represent the null value. (n = 3,967).

**
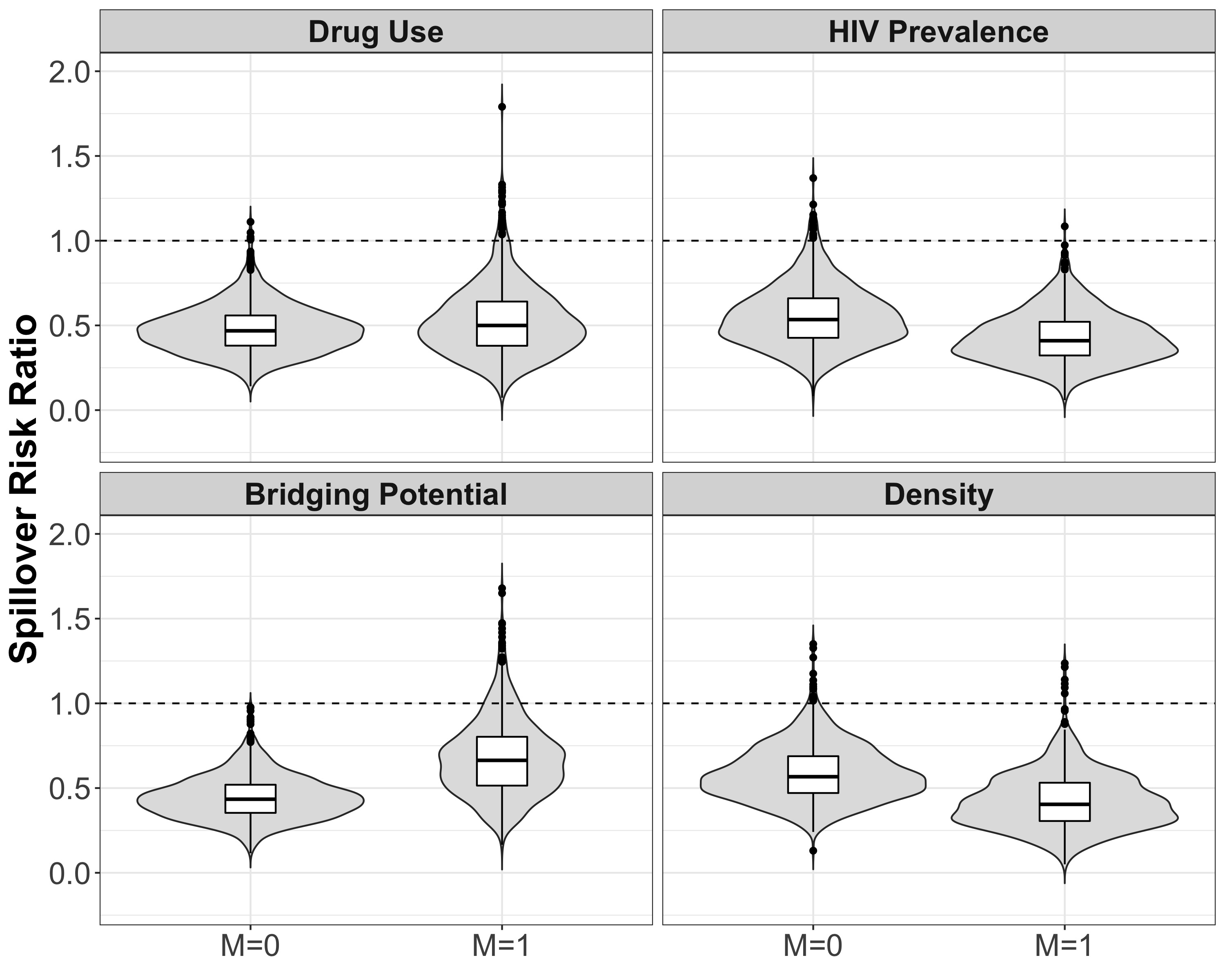
**

**Supplementary Figure S5.** Estimated risk difference spillover effects of PrEP on cumulative incidence of HIV by four modifiers (M = 1 if prevalence above median vs. M = 0 at or below median) in two-stage randomized designs of a pre-exposure prophylaxis (PrEP) intervention with 70% coverage in an agent-based model representing men who have sex with men, Atlanta metropolitan area, Georgia, 2015-2017 and *PrEP discontinuation in each monthly interval set to 10%***.** Lines within boxes, median values; box borders, interquartile ranges (75th and 25th percentiles); bars, 90th and 10th percentiles; points, outliers. Shaded shape represented the distribution of estimates and dashed lines represent the null value. (n = 3,896).

**
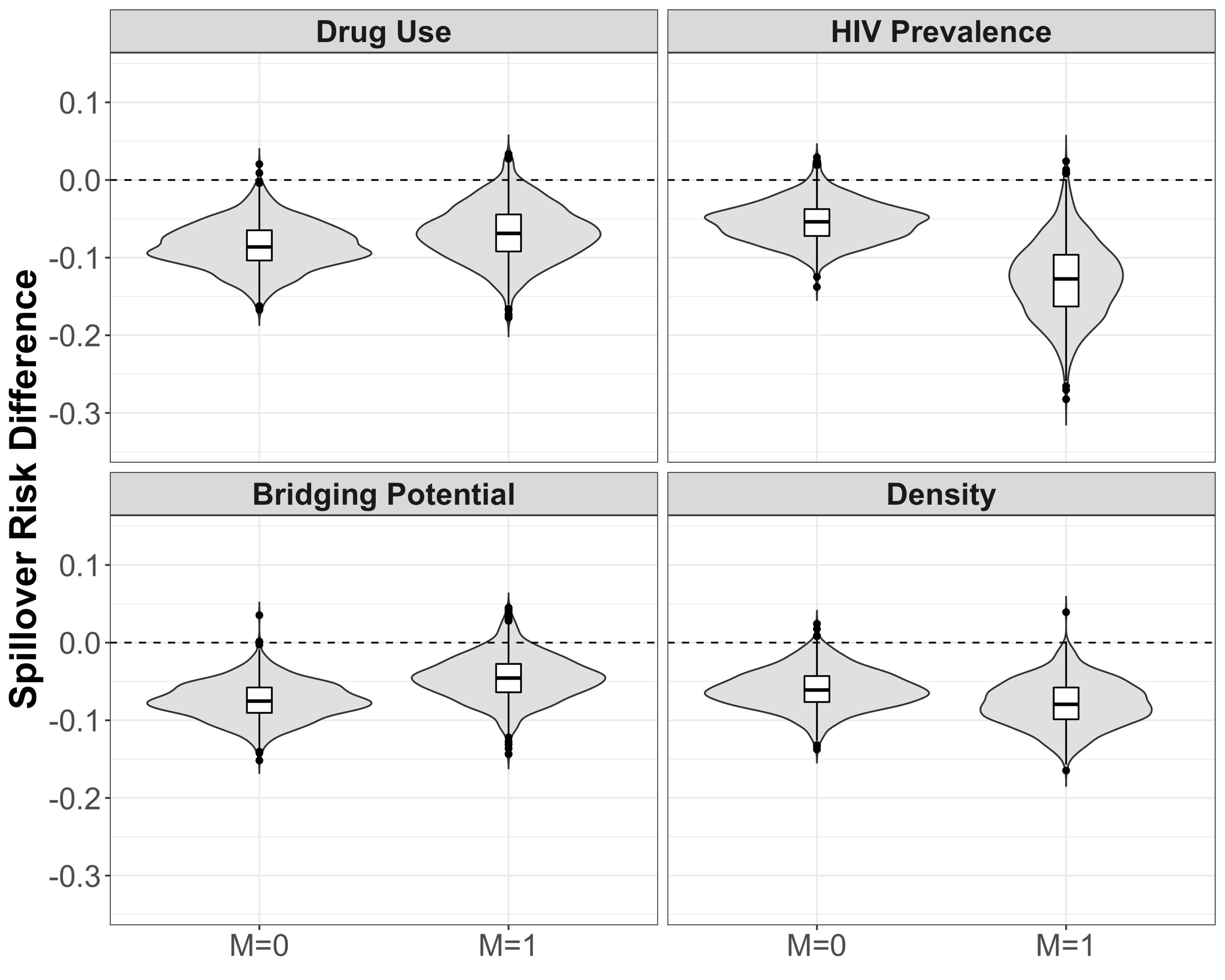
**

**Supplementary Figure S6.** Estimated risk ratio spillover effects of PrEP on cumulative incidence of HIV by four modifiers (M = 1 if prevalence above median vs. M = 0 at or below median) in two-stage randomized designs of a pre-exposure prophylaxis (PrEP) intervention with 70% coverage in an agent-based model representing men who have sex with men, Atlanta metropolitan area, Georgia, 2015-2017 and *PrEP discontinuation in each monthly interval set to 10%*. Lines within boxes, median values; box borders, interquartile ranges (75th and 25th percentiles); bars, 90th and 10th percentiles; points, outliers. Shaded shape represented the distribution of estimates and dashed lines represent the null value. (n = 3,896).

**
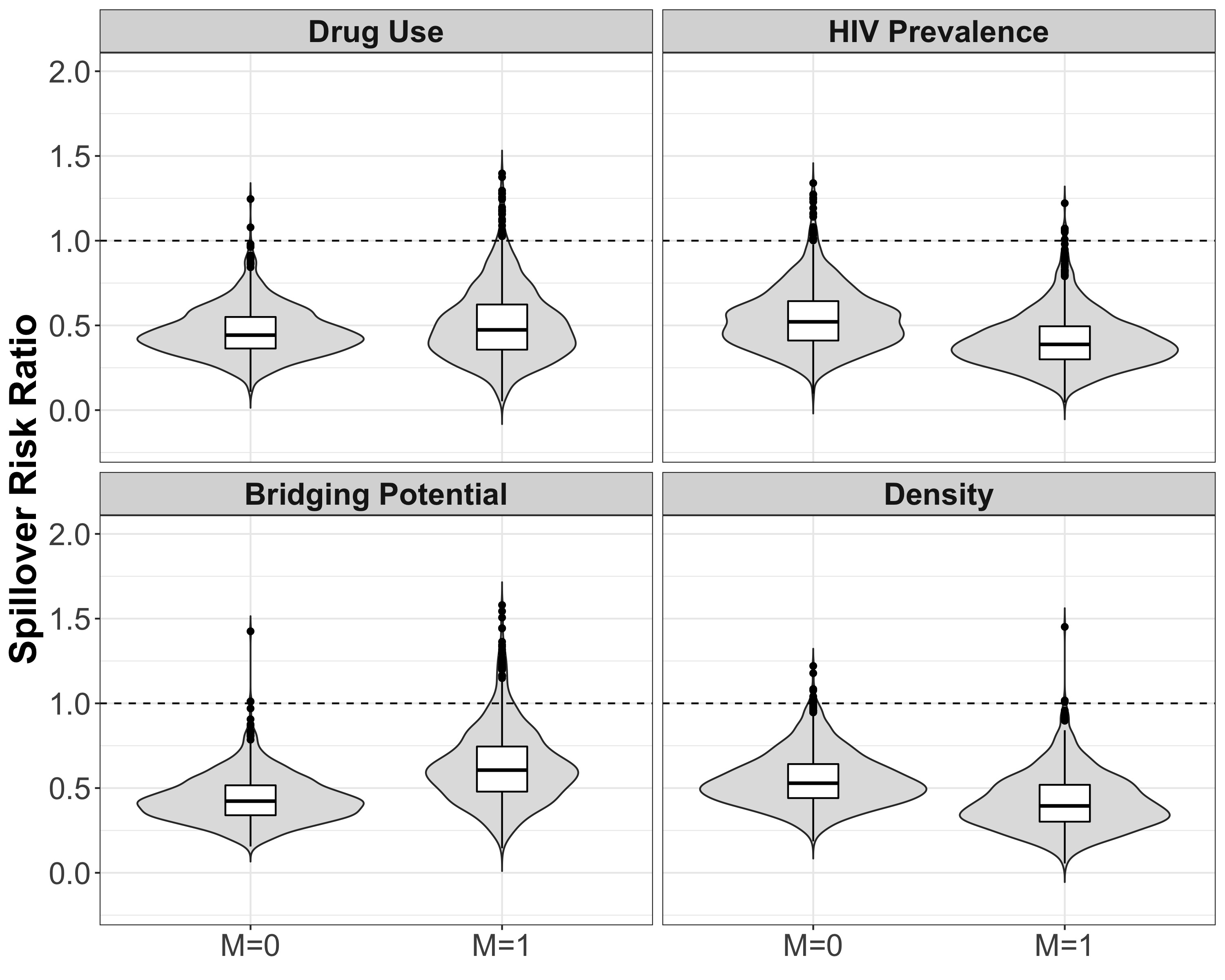
**

**References**

1. S. Bessey, Mary McGrath, Maximilian King, & Carolyn Park. (2020, November 10). marshall-lab/TITAN: v1.2.4 (Version v1.2.4).

2. Grant RM, Lama JR, Anderson PL, McMahan V, Liu AY, Vargas L, et al. Preexposure chemoprophylaxis for HIV prevention in men who have sex with men. *New England Journal of Medicine*. 2010;363(27):2587-99.

3. Halloran ME, Auranen K, Baird S, Basta NE, Bellan SE, Brookmeyer R, et al. Simulations for designing and interpreting intervention trials in infectious diseases. *BMC Medicine*. 2017;15(1):223.

4. Marshall BD, Goedel WC, King MR, Singleton A, Durham DP, Chan PA, Townsend JP, Galvani AP. Potential effectiveness of long-acting injectable pre-exposure prophylaxis for HIV prevention in men who have sex with men: a modelling study. *The Lancet HIV*. 2018;5(9):e498-e505.

5. Goedel WC, King MR, Lurie MN, Nunn AS, Chan PA, Marshall BD. Effect of racial inequities in pre-exposure prophylaxis use on racial disparities in HIV incidence among men who have sex with men: a modeling study. *Journal of Acquired Immune Deficiency Syndromes*. 2018;79(3):323-9.

6. Buchanan AL, Bessey S, Goedel WC, King M, Murray EJ, Friedman S, Halloran ME, Marshall BD. Spillover Effects in Agent Based Models: A Potential Outcomes Framework and Application to Inform Pre-Exposure Prophylaxis Coverage Levels for HIV Prevention. *American Journal of Epidemiology*. 2021 May;190(5):939-48.

7. Goedel WC, Bessey S, Lurie MN, Biello KB, Sullivan PS, Nunn AS, et al. Projecting the impact of equity-based pre-exposure prophylaxis implementation on racial disparities in HIV incidence among men who have sex with men. *AIDS*. 2020;34(10):1509-1517.

8. Grey JA, Bernstein KT, Sullivan PS, Purcell DW, Chesson HW, Gift TL, et al. Estimating the population sizes of men who have sex with men in US states and counties using data from the American Community Survey. *JMIR Public Health and Surveillance*. 2016;2(1):e5365.

9. Oster AM, Sternberg M, Lansky A, Broz D, Wejnert C, Paz-Bailey G. Population size estimates for men who have sex with men and persons who inject drugs. *Journal of Urban Health*. 2015;92(4):733-43.

10. Georgia Department of Public Health. HIV surveillance summary – Georgia, 2017. Atlanta, GA: Georgia Department of Public Health. Available at https://dph.georgia.gov/adult-core-hivaidssurveillance; 2019.

11. Sullivan PS, Rosenberg ES, Sanchez TH, Kelley CF, Luisi N, Cooper HL, et al. Explaining racial disparities in HIV incidence in black and white men who have sex with men in Atlanta, GA: A prospective observational cohort study. *Annals of Epidemiology*. 2015;25(6):445-54.

12. Hernández-Romieu AC, Sullivan PS, Rothenberg R, Grey J, Luisi N, Kelley CF, et al. Heterogeneity of HIV prevalence among the sexual networks of Black and White MSM in Atlanta: illuminating a mechanism for increased HIV risk for young Black MSM. *Sexually Transmitted Diseases*. 2015;42(9):505-512.

13. Millett GA, Peterson JL, Flores SA, Hart TA, Jeffries 4th WL, Wilson PA, et al. Comparisons of disparities and risks of HIV infection in black and other men who have sex with men in Canada, UK, and USA: a meta-analysis. *The Lancet*. 2012;380(9839):341-8.

14. Goodreau SM, Rosenberg ES, Jenness SM, Luisi N, Stansfield SE, Millett GA, et al. Sources of racial disparities in HIV prevalence in men who have sex with men in Atlanta, GA, USA: a modelling study. *The Lancet HIV*. 2017;4(7):e311-e20.

15. White D, Grey JA, Gorbach PM, Rothenberg RB, Sullivan PS, Rosenberg ES. Racial differences in partnership attributes, typologies, and risk behaviors among men who have sex with men in Atlanta, Georgia. *Archives of Sexual Behavior*. 2017;46(4):961-75.

16. Chan PA, Rose J, Maher J, Benben S, Pfeiffer K, Almonte A, et al. A latent class analysis of risk factors for acquiring HIV among men who have sex with men: Implications for implementing pre-exposure prophylaxis programs. *AIDS Patient Care STDs*. 2015;29(11):597-605.

17. Amirkhanian YA, Kelly JA, Kabakchieva E, Kirsanova AV, Vassileva S, Takacs J, et al. A randomized social network HIV prevention trial with young men who have sex with men in Russia and Bulgaria. *AIDS*. 2005;19(16):1897-905.

18. Walsh T, Schneider JA, Ardestani BM, Young LE. Individual and social network structure characteristics associated with peer change agent engagement and impact in a PrEP intervention. *AIDS and Behavior*. 2020; 24(12):3385-3394.

19. Wall KM, Stephenson R, Sullivan PS. Frequency of sexual activity with most recent male partner among young, Internet-using men who have sex with men in the United States. *Journal of Homosexuality*. 2013;60(10):1520-38.

20. Pagkas-Bather J, Young LE, Chen Y-T, Schneider JA. Social network interventions for HIV transmission elimination. *Current HIV/AIDS Reports*. 2020; 17(5):450-457.

21. Hernández-Romieu AC, Siegler AJ, Sullivan PS, Crosby R, Rosenberg ES. How often do condoms fail? A cross-sectional study exploring incomplete use of condoms, condom failures and other condom problems among black and white MSM in southern USA. *Sexually Transmitted Infections*. 2014;90(8):602-7.

22. Rendina HJ, Moody RL, Ventuneac A, Grov C, Parsons JT. Aggregate and event-level associations between substance use and sexual behavior among gay and bisexual men: comparing retrospective and prospective data. *Drug and Alcohol Dependence*. 2015;154:199-207.

23. Patel P, Borkowf CB, Brooks JT, Lasry A, Lansky A, Mermin J. Estimating per-act HIV transmission risk: a systematic review. *AIDS*. 2014;28(10):1509-1519.

24. Anderson PL, Glidden DV, Liu A, Buchbinder S, Lama JR, Guanira JV, et al. Emtricitabine-tenofovir concentrations and pre-exposure prophylaxis efficacy in men who have sex with men. *Science Translational Medicine*. 2012;4(151):151ra25-ra25.

25. Kelley CF, Rosenberg ES, O'Hara BM, Frew PM, Sanchez T, Peterson JL, et al. Measuring population transmission risk for HIV: an alternative metric of exposure risk in men who have sex with men (MSM) in the US. *PloS One*. 2012;7(12):e53284.

26. Li Z, Purcell DW, Sansom SL, Hayes D, Hall HI. Vital signs: HIV transmission along the continuum of care—United States, 2016. *Morbidity and Mortality Weekly Report.* 2019;68(11):267-272.

27. Singh S, Bradley H, Hu X, Skarbinski J, Hall HI, Lansky A. Men living with diagnosed HIV who have sex with men: progress along the continuum of HIV care—United States, 2010. *Morbidity and Mortality Weekly Report*. 2014;63(38):829-833.

28. Sterne JA, Hernán MA, Ledergerber B, Tilling K, Weber R, Sendi P, et al. Long-term effectiveness of potent antiretroviral therapy in preventing AIDS and death: a prospective cohort study. *The Lancet*. 2005;366(9483):378-84.

29. Chan PA, Mena L, Patel R, Oldenburg CE, Beauchamps L, Perez‐Brumer AG, et al. Retention in care outcomes for HIV pre‐exposure prophylaxis implementation programmes among men who have sex with men in three US cities. *J Int AIDS Soc*. 2016;19(1): 20903.

30. Eaton LA, Driffin DD, Bauermeister J, Smith H, Conway-Washington C. Minimal awareness and stalled uptake of pre-exposure prophylaxis (PrEP) among at risk, HIV-negative, black men who have sex with men. *J AIDS Patient Care STDs*. 2015;29(8):423-9.

31. Keen P, Bavinton BR. Could disparities in PrEP uptake limit the public health benefit? *The Lancet Public Health*. 2020;5(9):e467.

32. Grov C, Rendina HJ, John SA, Parsons JT. Determining the roles that club drugs, marijuana, and heavy drinking play in PrEP medication adherence among gay and bisexual men: implications for treatment and research. *AIDS and Behavior*. 2019;23(5):1277-86.

33. McKay MD, Beckman RJ, Conover WJ. A comparison of three methods for selecting values of input variables in the analysis of output from a computer code. *Technometrics*. 2000;42(1):55-61.

34. Van Rossum G, Drake FL Jr. *Python Reference Manual*. Amsterdam, the Netherlands: Center for Mathematics and Computer Science; 1995.28.

35. Walt Svd, Colbert SC, Varoquaux G. The NumPy array: a structure for efficient numerical computation. *Computing in Science & Engineering*. 2011;13(2):22-30.

36. Hagberg AA, Schult DA, Swart PJ. Exploring network structure, dynamics, and function using NetworkX. In: Varoquaux G, Vaught T, Millman J, eds. Proceedings of the 7th Python in Science Conference (SciPy 2008)—Austin, Texas, June 28–July 3, 2008. Austin, TX: SciPy; 2008:11–15. http://conference.scipy.org/proceedings/scipy2008/. Accessed May 28, 2019.30.

37. R Core Team. *R: A Language and Environment for Statistical Computing*. Vienna, Austria: R Foundation for Statistical Computing; 2014. http://www.R-project.org. Accessed July 9, 2019.35.

38. Wickham H. ggplot2: Elegant Graphics for Data Analysis. New York, NY: Springer Publishing Company; 2009.

39. Basse G, Feller A. Analyzing two-stage experiments in the presence of interference. *Journal of the American Statistical Association*. 2018;113(521):41-55.

40. Knol MJ, VanderWeele TJ. Recommendations for presenting analyses of effect modification and interaction. *International Journal of Epidemiology*. 2012;41(2):514-20.

41. Sobel ME. What do randomized studies of housing mobility demonstrate? Causal inference in the face of interference. *Journal of the American Statistical Association*. 2006;101(476):1398-407.

42. Hudgens MG, Halloran ME. Toward causal inference with interference. *Journal of the American Statistical Association*. 2008;103(482):832-42.

43. Ogburn EL, VanderWeele TJ. Causal diagrams for interference. *Statistical Science*. 2014;29(4):559-78.

44. Westreich D, Cole SR. Invited commentary: positivity in practice. *American Journal of Epidemiology*. 2010;171(6):674-7.

45. Pearl J. Brief Report: On the Consistency Rule in Causal Inference:" Axiom, Definition, Assumption, or Theorem?". *Epidemiology*. 2010:872-5.

46. VanderWeele TJ. Concerning the consistency assumption in causal inference. *Epidemiology*. 2009;20(6):880-3.

47. Burt RS. The social structure of competition. *Networks in the Knowledge Economy*. 2003;13:57-91.

48. Rothenberg RB, Potterat JJ, Woodhouse DE, Muth SQ, Darrow WW, Klovdahl AS. Social network dynamics and HIV transmission. *AIDS*. 1998;12(12):1529-36.

49. Valente TW, Fujimoto K. Bridging: locating critical connectors in a network. *Social Networks*. 2010;32(3):212-20.

50. Hong G, Raudenbush SW. Evaluating kindergarten retention policy: A case study of causal inference for multilevel observational data. *Journal of the American Statistical Association*. 2006;101(475):901-10.

51. Newman M (2018). Networks. Oxford: Oxford University Press.

52. Burchard J, Cornwell B. Structural holes and bridging in two-mode networks. *Social Networks*. 2018;55:11-20.

53. Schafer MH, Upenieks L, DeMaria J. Do Older Adults with HIV Have Distinctive Personal Networks? Stigma, Network Activation, and the Role of Disclosure in South Africa. *AIDS and Behavior*. 2021;25(5):1560-72.

54. United States Census Bureau. Community Facts - City of Atlanta, Georgia [Web]. 2010 [cited 2017 March 11]. Available from: <https://factfinder.census.gov/faces/tableservices/jsf/pages/productview.xhtml?src=CF>.

55. Liu AY, Cohen SE, Vittinghoff E, Anderson PL, Doblecki-Lewis S, Bacon O, et al. Preexposure prophylaxis for HIV infection integrated with municipal-and community-based sexual health services. *JAMA Internal Medicine*. 2016;176(1):75-84.
